# Impact of adjuvant chemotherapy on T1N0M0 breast cancer patients: a propensity score matching study based on SEER database and external cohort

**DOI:** 10.1186/s12885-022-09952-z

**Published:** 2022-08-08

**Authors:** Kaiwen Shen, Longdi Yao, Jingyuan Zhu, Ximing Gu, Jie Wang, Wei Qian, Zhijian Zheng, Deyuan Fu, Song Wu

**Affiliations:** 1 Department of General Surgery, Traditional Chinese Medicine Hospital of Kunshan, Suzhou, 215000 Jiangsu China; 2 Department of General Surgery, Changxing Hospital of Traditional Chinese Medicine, Huzhou, 313100 Zhejiang China; 3 Nanjing Medical University, Nanjing, 211166 Jiangsu China; 4 Department of General Surgery, The First People’s Hospital of Wenling, Wenling, 317500 Zhejiang China; 5 Department of Thyroid and Breast Surgery, Northern Jiangsu People’s Hospital, Yangzhou University Medical Academy, Guangling District, Nantong Xi Road, Yangzhou, 225001 Jiangsu China; 6 Department of Thyroid and Breast Surgery, The First People’s Hospital of Wenling, Chuanan Nan Road, Chengxi Street, Wenling, 317500 Zhejiang China

**Keywords:** Adjuvant chemotherapy, Breast neoplasms, Small tumor, Prognosis, Molecular subtype, Lymph node–negative

## Abstract

**Background:**

There is no clear consensus on the benefits of adjuvant chemotherapy for tumor-node-metastasis (TNM) stage T1 (T1N0M0) breast cancer (BC). Our study investigated the effects of adjuvant chemotherapy on T1N0M0 BC patients.

**Methods:**

Seventy-five thousand one hundred thirty-nine patients diagnosed with T1N0M0 BC were selected from the Surveillance, Epidemiology, and End Results (SEER) database. Multivariate Cox analyses were performed to investigate the effects of adjuvant chemotherapy on T1a, T1b, and T1cN0M0 BC, including various tumor grades, and four molecular subtypes. Propensity score matching (PSM) was used to eliminate confounding factors and further compare the results between adjuvant chemotherapy and no adjuvant chemotherapy. Additionally, 545 T1N0M0 BC patients treated at the Northern Jiangsu People’s Hospital were included as an independent external validation cohort. Univariate and multivariate Cox analyses were used to confirm the effects of adjuvant chemotherapy in T1a, T1b, and T1cN0M0 BC. Survival curves for the different tumor grades and molecular subtypes were plotted using the Kaplan–Meier method.

**Results:**

Adjuvant chemotherapy demonstrated a statistically significant improvement in overall survival (OS) in T1b and T1c BC, but not in T1a BC. Within T1b BC, adjuvant chemotherapy was found to have effects on grade III, and hormone receptor + (HoR +)/human epidermal growth factor receptor 2 + (HER2 +), HoR-/HER2 + , and HoR-/HER2- molecular subtypes, respectively. Adjuvant chemotherapy was beneficial to OS for grade II/III and T1c BC. Identical results were obtained after PSM. We also obtained similar results with external validation cohort, except that adjuvant chemotherapy made a difference in grade II and T1b BC of the external validation dataset.

**Conclusions:**

Partial T1N0M0 BC patients with grade III T1bN0M0, patients with tumor grade II and III T1cN0M0, and excluding those with HoR + /HER2- subtype tumors, could obtain OS benefits from adjuvant chemotherapy.

**Supplementary Information:**

The online version contains supplementary material available at 10.1186/s12885-022-09952-z.

## Background

Breast cancer (BC) is the most commonly occurring malignancy in women and the leading cause of cancer-related death among women worldwide [1]. The incidence of early-stage BC has increased over the recent decades due to the widespread use of advanced diagnostic imaging and longer life expectancy [2–5]. Small tumors without lymph node infiltration have been given much attention by physicians. These patients are considered to have a good prognosis, even though they undergo surgery without adjuvant therapy. T1N0M0 BC includes tumors smaller than 2 cm without node involvement. These tumors are subdivided into three groups: T1a (≤ 0.5 cm), T1b (> 0.5 cm but ≤ 1.0 cm), and T1c (> 1.0 cm but ≤ 2.0 cm) [6]. According to the seventh edition of the American Joint Committee on Cancer, T1N0M0 BC has been reported to have a relatively low risk of death and recurrence [7, 8].

BC is a heterogeneous disease with distinct response to therapeutics, and consists of four different molecular subtypes, namely, luminal A, luminal B, HER2 + , and triple-negative breast cancer (TNBC) [9]. Considering the results of international reviews, adjuvant therapy for BC can reduce deaths by approximately 25% across all the risk groups [10]. Adjuvant systematic therapy includes chemotherapy, endocrine therapy, and targeted therapy. Although adjuvant chemotherapy is an effective treatment, it is known to cause short-term and long-term side effects that are toxic and could result in death [11]. Patients with early-stage BC have been reported to achieve only a small absolute percentage of survival benefit from adjuvant chemotherapy [12]. Therefore, it is crucial to identify the patients with early-stage BC that may benefit from adjuvant chemotherapy. When considering adjuvant chemotherapy, it is important to weigh the possible risks against the benefits. Adjuvant chemotherapy can reduce the risk of tumor recurrence and death, but it also increases the damage caused by its toxic side-effects and increases the medical expenses. Nevertheless, without further evidence, the possible benefits of adjuvant chemotherapy for early-stage BC are controversial. Therefore, our study aimed to evaluate the effects of adjuvant chemotherapy on the overall survival (OS) of T1N0M0 BC patients.

## Materials and methods

### Data acquisition and patient selection

The Surveillance, Epidemiology, and End Results (SEER) database was established in 1973 by the National Cancer Institute and is one of the largest tumor registration database. Data from 18 registries of the SEER program (2010 to 2014) was used to identify T1N0M0 female BC patients. Breast cancer patients that met the following criteria were excluded: (a) did not undergo surgery; (b) had a history of ductal carcinoma in situ or lobular carcinoma in situ; (c) had a history of other malignancy /chronic diseases; (d) participated for < 3 months of follow-up; and (e) had incomplete or missing clinicopathological data.

We collected the data of 75,139 early-stage BC patients from the SEER database. The same inclusion and exclusion criteria were applied to the external validation cohort, consisting of patients treated at the Northern Jiangsu People’s Hospital from 2010 to 2015. Finally, 545 female T1N0M0 BC patients were included in the study from the external validation cohort.

The following data of each patient were gathered: patient number, age, death status (yes/no), follow-up time, tumor size, grade, estrogen receptor (ER) status, progesterone receptor (PR) status, human epidermal growth factor receptor‐2 (HER‐2) status, surgical method, history of adjuvant chemotherapy, and history of radiation therapy. Because chemotherapy-related toxicities could lead to death, we chose OS as the endpoint instead of BC-specific survival [13]. An age of 60 years was chosen as the threshold for distinguishing young and old patients [14].

### SEER methods

A cohort of 75,139 eligible early-stage BC patients was identified from the SEER database. We conducted a descriptive analysis of the baseline clinical features of eligible patients and used the chi-square test to compare the characteristics of patients among the three groups. Multivariate Cox regression analysis was performed to explore whether adjuvant chemotherapy was a prognostic factor for T1a, T1b, and T1c BC patients. We also performed a multivariate Cox regression analysis of diverse tumor grades and molecular subtypes. To further confirm the specific role of adjuvant chemotherapy for patients in the three groups and four molecular subtypes, a multivariate Cox regression analysis of tumor grades was performed. Furthermore, propensity score matching (PSM) was performed to balance the disparities between adjuvant chemotherapy and no adjuvant chemotherapy. The variables considered in the PSM analysis for adjuvant chemotherapy status were tumor grade, surgery type, radiation record, molecular subtype, and age. Following PSM, multivariate Cox regression analysis was repeated in order to obtain more accurate results, and to assess the usefulness of adjuvant chemotherapy for the different tumor grades and molecular subtypes of the three groups.

### External validation methods

A total of 545 T1N0M0 female BC patients were included in the external validation cohort from the Northern Jiangsu People’s Hospital. Because of the lack of data, we used the univariate Cox regression analysis to identify the significant variables and remove some inconsequential parameters. These meaningful indicators were incorporated into the multivariate Cox regression analysis to further evaluate the three groups. In each of the three groups, Kaplan–Meier survival curves of the tumor grades and molecular subtypes were plotted and the estimated log-rank test results were used to compare the control group (no adjuvant chemotherapy) and the experimental group (adjuvant chemotherapy).

### Statistical methods

Data analyses were performed using the R software version 3.5.1 (R Foundation for Statistical Computing, Vienna, Austria) and SPSS version 19.0 (IBM Corporation, Armonk, NY, USA). PSM was calculated using multivariate logistic regression, and the propensity score was constructed using 1:1 nearest-neighbor matching within calipers (0.005), without replacement [15]. Two-tailed test with a *p*-value (*P*) < 0.05 was considered statistically significant.

## Results

### SEER results

The median follow-up time was 51 months, as calculated by the reverse Kaplan–Meier method. As shown in Table S1, a total of 75,139 T1N0M0 BC patients were divided into three groups: T1a (*n* = 10,073); T1b (*n* = 24,951); and T1c (*n* = 40,115). T1cN0M0 BC patients tended to have worse differentiated tumor grades than the other groups and often received adjuvant chemotherapy. There were notable statistical differences between the groups (*P* < 0.01). The outcomes of multivariate Cox proportional hazard analyses of T1, T1a, T1b, and T1c are presented in Table S2. Adjuvant chemotherapy was effective for T1b (hazard ratio (HR), 0.72; 95% confidence interval (CI), 0.59–0.88; *P* < 0.0001) and T1c (HR, 0.54; 95% CI, 0.48–0.60; *P* < 0.0001). Compared to the other subtypes, HoR + /HER2- BC subtype had a better prognosis with T1b and T1c. Patients treated with radiotherapy had more favorable survival rates (*P* < 0.0001). Multivariate Cox analyses (Table S3) showed that patients that underwent breast-conserving surgery combined with radiotherapy experienced a longer OS than patients that underwent breast-conserving surgery or total mastectomy or modified radical mastectomy alone (*P* < 0.0001). As expected, larger tumors were strong predictors of worse OS. Multivariate Cox regression analysis of the four molecular subtypes and tumor grades of T1a are presented in Tables S4 and S5. Adjuvant chemotherapy did not have a role in molecular subtypes HoR + /HER2- (*P* = 0.11), HoR + /HER2 + (*P* = 0.36), HoR-/HER2 + (*P* = 0.22), and HoR-/HER2- (*P* = 0.20) and tumor grades I (*P* = 0.78), II (*P* = 0.23), and III (*P* = 0.68). Multivariate Cox regression analysis of the four molecular subtypes and tumor grades of T1b are presented in Tables S6 and S7. There were no survival benefits with adjuvant chemotherapy in T1bN0M0 BC patients with HoR + /HER2- (*P* = 0.67), grade I (*P* = 0.41), and grade II (*P* = 0.11) tumors. There were statistically significant survival benefits with adjuvant chemotherapy in T1bN0M0 BC patients with HoR + /HER2 + (HR, 0.41; 95% CI, 0.25–0.69; *P* < 0.0001), HoR-/HER2 + (HR, 0.50; 95% CI, 0.25–1.02; *P* = 0.01), HoR-/HER2- (HR, 0.53; 95% CI, 0.36–0.78; *P* < 0.0001), and grade III (HR, 0.52; 95% CI, 0.38–0.70; *P* < 0.0001) tumors, as compared to patients that did not receive any adjuvant chemotherapy. T1c patients that received adjuvant chemotherapy appeared to have OS benefits compared to their counterparts, except for those with grade I (*P* = 0.10) (Tables S8 and S9). Multivariate Cox regression analyses of tumor grades of the T1a group and molecular subtype subgroups are presented in Tables S10, S11, S12, and S13. Not surprisingly, adjuvant chemotherapy did not improve the OS of T1a patients. However, adjuvant chemotherapy decreased the survival time for grade II (HR, 3.23; 95% CI, 1.81–5.78; *P* < 0.0001) and grade III (HR, 3.26; 95% CI, 1.25–8.48; *P* = 0.02) T1a patients with HoR + /HER2- subtype. Adjuvant chemotherapy led to accelerated death in grade I T1b patients with HoR + /HER2- (HR, 1.62; 95% CI, 1.02–2.57; *P* = 0.04) (Tables S14, S15, S16, and S17). Adjuvant chemotherapy also significantly enhanced the OS of grade III (HR, 0.36; 95% CI, 0.16–0.80; P = 0.01) T1b patients with HoR + /HER2 + , HoR-/HER2 + (HR, 0.34; 95% CI, 0.14–0.84; *P* = 0.02), and HoR-/HER2- (HR, 0.54; 95% CI, 0.34–0.85; *P* = 0.01). However, adjuvant chemotherapy was not useful for grade I T1c patients with HoR + /HER2- (*P* = 0.20), HoR + /HER2 + (*P* = 0.28), HoR-/HER2 + (*P* = 0.41), and HoR-/HER2- (*P* = 0.67) (Tables S18, S19, S20, and S21). Tables 1, 2 and 3 summarize the association of adjuvant chemotherapy with other variables. The results of the adjuvant chemotherapy group were clearly contrary to that of the no adjuvant chemotherapy group of the entire cohort (*P* < 0.01). After PSM, the parameters of the above two groups were similar in the matched cohort. Consequently, these matched data were used for further analyses and validation. In the matched cohort, adjuvant chemotherapy was statistically significant for T1b (HR, 0.44; 95% CI, 0.38–0.51; *P* < 0.001) and T1c (HR, 0.47; 95% CI, 0.41–0.54; *P* < 0.001) (Table 4). Similarly, T1a patients of the matched cohort (Tables 5 and 6) with tumor grades I (*P* = 0.27), II (*P* = 0.99), and III (*P* = 0.57) and molecular subtypes HoR + /HER2- (*P* = 0.66), HoR + /HER2 + (*P* = 0.20), HoR/HER2 + (*P* = 0.27), and HoR-/HER2- (*P* = 0.48) did not obtain OS benefit from adjuvant chemotherapy. Furthermore, T1b patients with HoR + /HER2 + (HR, 0.38; 95% CI, 0.21–0.66; *P* < 0.01), HoR-/HER2 + (HR, 0.51; 95% CI, 0.23–1.15; *P* = 0.01), HoR-/HER2- (HR, 0.44; 95% CI, 0.28–0.67; *P* < 0.0001) and grade III (HR, 0.51; 95% CI, 0.36–0.72; *P* < 0.0001), that received adjuvant chemotherapy, showed OS benefits (Tables 7 and 8). T1c patients that received adjuvant chemotherapy, appeared to obtain OS benefits compared to their counterparts, except for those with grade I (*P* = 0.33) of the matched cohort (Tables 9 and 10).

**Table 1:** Characteristics of T1a patients with or without chemotherapy in the whole cohort and matched cohorts

Characteristic	T1a: Whole cohort [cases (%)]			T1a: Matched cohort [cases (%)]		
	Chemotherapy		*P*-value	Chemotherapy		*P*-value
	No	Yes		No	Yes	
**GRADE**			< 0.01			0.87
I	4254 (46.11%)	128 (15.09%)		135 (16.03%)	128 (15.20%)	
II	3906 (42.34%)	368 (43.40%)		358 (42.52%)	366 (43.47%)	
III	1065 (11.54%)	352 (41.51%)		349 (41.45%)	348 (41.33%)	
**SURGERY**			< 0.01			0.94
Breast-conserving	6054 (65.63%)	400 (47.17%)		393 (46.67%)	400 (47.51%)	
Total mastectomy	2603 (28.22%)	353 (41.63%)		355 (42.16%)	349 (41.45%)	
Modified radical mastectomy	568 (6.16%)	95 (11.20%)		94 (11.16%)	93 (11.05%)	
**RADIATION**			< 0.01			0.66
No	4016 (43.53%)	466 (54.95%)		475 (56.41%)	466 (55.34%)	
Yes	5209 (56.47%)	382 (45.05%)		367 (43.59%)	376 (44.66%)	
**SUBTYPE**			< 0.01			0.86
HoR + /HER2-	7766 (84.18%)	311 (36.67%)		323 (38.36%)	311 (36.94%)	
HoR + /HER2 +	621 (6.73%)	239 (28.18%)		224 (26.60%)	239 (28.38%)	
HoR-/HER2 +	302 (3.27%)	138 (16.27%)		138 (16.39%)	135 (16.03%)	
HoR-/HER2-	536 (5.81%)	160 (18.87%)		157 (18.65%)	157 (18.65%)	
**AGE (year)**			< 0.01			0.71
< 60	3818 (41.39%)	575 (67.81%)		580 (68.88%)	573 (68.05%)	
≥ 60	5407 (58.61%)	273 (32.19%)		262 (31.12%)	269 (31.95%)	

Abbreviations: *HoR* hormone receptor, *HER‐2* human epidermal growth factor receptor‐2

**Table 2:** Characteristics of T1b patients with or without chemotherapy in the whole cohort and matched cohorts

Characteristic	T1b: whole cohort [cases (%)]			T1b: Matched cohort [cases (%)]		
	Chemotherapy		*P*-value	Chemotherapy		*P*-value
	No	Yes		No	Yes	
**GRADE**			< 0.01			0.83
I	9790 (45.96%)	466 (12.77%)		443 (16.07%)	455 (16.50%)	
II	9455 (44.38%)	1442 (39.53%)		1197 (43.42%)	1176 (42.66%)	
III	2058 (9.66%)	1740 (47.70%)		1117 (40.52%)	1126 (40.84%)	
**SURGERY**			< 0.01			0.92
Breast-conserving	15,453 (72.54%)	2235 (61.27%)		1676 (60.79%)	1665 (60.39%)	
Total mastectomy	4615 (21.66%)	1147 (31.44%)		869 (31.52%)	883 (32.03%)	
Modified radical mastectomy	1235 (5.80%)	266 (7.29%)		212 (7.69%)	209 (7.58%)	
**RADIATION**			< 0.01			0.77
No	7997 (37.54%)	1569 (43.01%)		1238 (44.90%)	1227 (44.50%)	
Yes	13,306 (62.46%)	2079 (56.99%)		1519 (55.10%)	1530 (55.50%)	
**SUBTYPE**			< 0.01			0.96
HoR + /HER2-	19,702 (92.48%)	1509 (41.37%)		1512 (54.84%)	1508 (54.70%)	
HoR + /HER2 +	712 (3.34%)	876 (24.01%)		538 (19.51%)	548 (19.88%)	
HoR-/HER2 +	147 (0.69%)	339 (9.29%)		139 (5.04%)	144 (5.22%)	
HoR-/HER2-	742 (3.48%)	924 (25.33%)		568 (20.60%)	557 (20.20%)	
**AGE (year)**			< 0.01			0.83
< 60	7510 (35.25%)	2243 (61.49%)		1416 (51.36%)	1424 (51.65%)	
≥ 60	13,793 (64.75%)	1405 (38.51%)		1341 (48.64%)	1333 (48.35%)	

Abbreviations: *HoR* hormone receptor, *HER‐2* human epidermal growth factor receptor‐2

**Table 3:** Characteristics of T1c patients with or without chemotherapy in the whole cohort and matched cohorts

Characteristic	T1c: whole cohort [cases (%)]			T1c: Matched cohort [cases (%)]		
	Chemotherapy		*P*-value	Chemotherapy		*P*-value
	No	Yes		No	Yes	
**GRADE**			< 0.01			0.64
I	9648 (32.86%)	836 (7.77%)		777 (11.42%)	802 (11.79%)	
II	15,475 (52.70%)	3823 (35.55%)		3028 (44.52%)	2979 (43.80%)	
III	4239 (14.44%)	6094 (56.67%)		2997 (44.06%)	3021 (44.41%)	
**SURGERY**			< 0.01			0.61
Breast-conserving	19,489 (66.37%)	6389 (59.42%)		3956 (58.16%)	3974 (58.42%)	
Total mastectomy	7640 (26.02%)	3379 (31.42%)		2174 (31.96%)	2190 (32.20%)	
Modified radical mastectomy	2233 (7.61%)	985 (9.16%)		672 (9.88%)	638 (9.38%)	
**RADIATION**			0.96			0.41
No	12,848 (43.76%)	4708 (43.78%)		3307 (48.62%)	3259 (47.91%)	
Yes	16,514 (56.24%)	6045 (56.22%)		3495 (51.38%)	3543 (52.09%)	
**SUBTYPE**			< 0.01			0.93
HoR + /HER2-	26,924 (91.70%)	4984 (46.35%)		4579 (67.32%)	4579 (67.32%)	
HoR + /HER2 +	973 (3.31%)	2324 (21.61%)		873 (12.83%)	891 (13.10%)	
HoR-/HER2 +	256 ( 0.87%)	769 (7.15%)		246 ( 3.62%)	250 (3.68%)	
HoR-/HER2-	1209 (4.12%)	2676 (24.89%)		1104 (16.23%)	1082 (15.91%)	
**AGE (year)**			< 0.01			0.76
< 60	10,350 (35.25%)	6891 (64.08%)		3645 (53.59%)	3663 (53.85%)	
≥ 60	19,012 (64.75%)	3862 (35.92%)		3157 (46.41%)	3139 (46.15%)	

Abbreviations: *HoR* hormone receptor, *HER‐2* human epidermal growth factor receptor‐2

**Table 4:** Multivariate Cox regression analyses of overall survival for T1a, T1b, and T1c breast cancer patients in the matched cohort

Variables	T1a		T1b		T1c	
	Multivariate Analysis		Multivariate Analysis		Multivariate Analysis	
	HR (95%CI)	*P*-value	HR (95%CI)	*P*-value	HR (95%CI)	*P*-value
**GRADE**						
I	reference		reference		reference	
II	2.17(0.98–4.83)	0.06	1.01(0.89–1.14)	0.92	1.25(0.95–1.63)	0.11
III	2.24(0.95–5.27)	0.07	1.19(0.99–1.43)	0.06	1.59(1.22–2.08)	< 0.01
**SURGERY**						
Breast-conserving	reference		reference		reference	
Total mastectomy	0.67(0.31–1.43)	0.30	0.74(0.63–0.88)	< 0.01	0.58(0.48–0.69)	< 0.0001
Modified radical mastectomy	0.26(0.08–0.85)	0.03	0.96(0.77–1.19)	0.70	0.63(0.50–0.80)	< 0.01
**RADIATION**						
No	reference				reference	
Yes	0.32(0.15–0.70)	< 0.01	0.72(0.59–0.88)	< 0.0001	0.39(0.33–0.47)	< 0.0001
**CHEMOTHERAPY**						
No	reference		reference		reference	
Yes	1.09(0.67–1.76)	0.73	0.44(0.38–0.51)	< 0.0001	0.47(0.41–0.54)	< 0.0001
**SUBTYPE**						
HoR + /HER2-	reference		reference		reference	
HoR + /HER2 +	0.35(0.16–0.75)	0.01	1.32(1.05–1.68)	0.02	1.31(1.09–1.57)	< 0.01
HoR-/HER2 +	0.70(0.35–1.38)	0.30	1.64(1.14–2.37)	0.01	1.41(1.06–1.86)	0.02
HoR-/HER2-	0.55(0.27–1.13)	0.10	1.65(1.33–2.05)	< 0.0001	1.83(1.57–2.14)	< 0.0001
**AGE (year)**						
< 60	reference		reference		reference	
≥ 60	3.22(1.95–5.31)	< 0.0001	3.72(3.17–4.36)	< 0.0001	3.08(2.65–3.58)	< 0.0001

Abbreviations: *HR* hazard ratio, *HoR* hormone receptor, *HER‐2* human epidermal growth factor receptor‐2

**Table 5:** Multivariate Cox regression analyses of overall survival for four molecular subtypes of T1a breast cancer patients in the matched cohort

**Variable**	HoR + /HER2-		HoR + /HER2 +		HoR-/HER2 +		HoR-/HER2-	
	Multivariate Analysis		Multivariate Analysis		Multivariate Analysis		Multivariate Analysis	
	HR (95%CI)	*P*-value	HR (95%CI)	*P*-value	HR (95%CI)	*P*-value	HR (95%CI)	*P*-value
**GRADE**								
I	reference		reference		reference		reference	
II	2.21(0.91–5.36)	0.08	-	-	0.55(0.07–4.62)	0.58	-	-
III	2.92(1.08–7.92)	0.04	-	-	0.35(0.04–3.24)	0.35	-	-
**SURGERY**								
Breast-conserving	reference		reference		reference		reference	
Total mastectomy	0.46(0.17–1.23)	0.12	22.06(2.09–232.47)	0.01	1.79(0.11–28.43)	0.68	0.44(0.11–1.85)	0.26
Modified radical mastectomy	0.29(0.07–1.24)	0.09	-	-	1.09(0.04–27.13)	0.96	-	-
**RADIATION**								
No	reference		reference		reference		reference	
Yes	0.20(0.07–0.58)	< 0.01	6.33(0.66–60.72)	0.11	1.41(0.09–21.27)	0.80	0.19(0.04–0.85)	0.03
**CHEMOTHERAPY**								
No	reference		reference		reference		reference	
Yes	2.25(1.09–4.62)	0.66	0.27(0.06–1.25)	0.10	0.51(0.15–1.70)	0.27	0.66(0.21–2.10)	0.48
**AGE (year)**								
< 60	reference		reference		reference		reference	
≥ 60	5.22(2.40–11.32)	< 0.0001	52.09(6.10–444.61)	< 0.01	1.31(0.37–4.67)	0.68	0.47(0.13–1.77)	0.27

Abbreviations: *HoR* hormone receptor, *HER2* human epidermal growth factor receptor‐2, *HR* hazard ratio

**Table 6:** Multivariate Cox regression analyses of overall survival for tumor grades of T1a breast cancer patients in the matched cohort

**Variable**	T1a: GRADEI		T1a: GRADEII		T1a: GRADEIII	
	**Multivariate Analysis**		**Multivariate Analysis**		**Multivariate Analysis**	
	HR (95%CI)	*P*-value	HR (95%CI)	*P*-value	HR (95%CI)	*P*-value
**SURGERY**						
Breast-conserving	reference		reference		reference	
Total mastectomy	0.36(0.03–3.94)	0.41	2.13(0.59–7.72)	0.25	0.52(0.14–1.90)	0.32
Modified radical mastectomy	0.54(0.03–10.37)	0.68	1.99(0.46–8.65)	0.36	0.29(0.05–1.78)	0.18
**RADIATION**						
No	reference		reference		reference	
Yes	-	-	1.78(0.52–6.12)	0.36	0.23(0.06–0.87)	0.03
**CHEMOTHERAPY**						
No	reference		reference		reference	
Yes	2.54(0.49–13.09)	0.27	1.00(0.51–1.94)	0.99	1.27(0.56–2.91)	0.57
**SUBTYPE**						
HoR + /HER2-	reference		reference		reference	
HoR + /HER2 +	-	-	0.22(0.06–0.75)	0.02	0.10(0.01–0.79)	0.03
HoR-/HER2 +	1.73(0.20–15.15)	0.62	1.00(0.43–2.33)	1.00	0.68(0.22–2.15)	0.52
HoR-/HER2-	-	-	1.13(0.44–2.93)	0.80	0.58(0.21–1.57)	0.28
**AGE (year)**						
< 60	reference		reference		reference	
≥ 60	0.31(0.03–3.20)	0.32	3.35(1.64–6.83)	< 0.01	2.89(1.25–6.68)	0.01

Abbreviations: *HR* hazard ratio, *HoR* hormone receptor, *HER2* human epidermal growth factor receptor‐2

**Table 7:** Multivariate Cox regression analyses of overall survival for four molecular subtypes of T1b breast cancer patients in the matched cohort

**Variable**	HoR + /HER2-		HoR + /HER2 +		HoR-/HER2 +		HoR-/HER2-	
	Multivariate Analysis		Multivariate Analysis		Multivariate Analysis		Multivariate Analysis	
	HR (95%CI)	*P*-value	HR (95%CI)	*P*-value	HR (95%CI)	*P*-value	HR (95%CI)	*P*-value
**GRADE**								
I	reference		reference		reference		reference	
II	0.82(0.52–1.29)	0.39	0.74(0.30–1.85)	0.52	0.83(0.10–6.79)	0.87	1.29(0.31–5.44)	0.73
III	0.93(0.58–1.48)	0.76	0.88(0.35–2.23)	0.79	0.87(0.11–6.77)	0.89	1.32(0.32–5.42)	0.70
**SURGERY**								
Breast-conserving	reference		reference		reference		reference	
Total mastectomy	1.14(0.61–2.11)	0.68	0.64(0.28–1.46)	0.29	0.28(0.09–0.84)	0.02	1.65(0.77–3.54)	0.20
Modified radical mastectomy	1.64(0.81–3.30)	0.17	1.05(0.34–3.23)	0.94	0.40(0.09–1.70)	0.21	1.79(0.72–4.44)	0.21
**RADIATION**								
No	reference		reference		reference		reference	
Yes	0.63(0.35–1.14)	0.13	0.59(0.27–1.27)	0.18	0.33(0.10–1.06)	0.06	0.93(0.45–1.93)	0.84
**CHEMOTHERAPY**								
No	reference		reference		reference		reference	
Yes	0.94(0.66–1.34)	0.74	0.38(0.21–0.66)	< 0.01	0.51(0.23–1.15)	0.01	0.44(0.28–0.67)	< 0.0001
**AGE (year)**								
< 60	reference		reference		reference		reference	
≥ 60	3.92(2.67–5.74)	< 0.0001	4.93(2.37–10.28)	< 0.0001	2.34(0.87–6.32)	0.09	3.34(1.81–6.15)	< 0.0001

Abbreviations: *HoR* hormone receptor, *HER2* human epidermal growth factor receptor‐2, *HR* hazard ratio

**Table 8:** Multivariate Cox regression analyses of overall survival for tumor grades of T1b breast cancer patients in the matched cohort

**Variable**	T1b: GRADEI		T1b: GRADEII		T1b: GRADEIII	
	**Multivariate Analysis**		**Multivariate Analysis**		**Multivariate Analysis**	
	HR (95%CI)	*P*-value	HR (95%CI)	*P*-value	HR (95%CI)	*P*-value
**SURGERY**						
Breast-conserving	reference		reference		reference	
Total mastectomy	0.88(0.35–2.19)	0.78	1.23(0.60–2.52)	0.58	0.91(0.50–1.66)	0.77
Modified radical mastectomy	2.34(0.79–6.96)	0.13	2.06(0.91–4.67)	0.08	0.71(0.32–1.58)	0.40
**RADIATION**						
No	reference		reference		reference	
Yes	0.55(0.23–1.34)	0.19	0.83(0.41–1.65)	0.59	0.76(0.43–1.36)	0.36
**CHEMOTHERAPY**						
No	reference		reference		reference	
Yes	0.83(0.45–1.52)	0.54	0.80(0.55–1.18)	0.26	0.51(0.36–0.72)	< 0.0001
**SUBTYPE**						
HoR + /HER2-	reference		reference		reference	
HoR + /HER2 +	1.68(0.77–3.64)	0.19	1.10(0.68–1.78)	0.70	1.25(0.74–2.10)	0.41
HoR-/HER2 +	1.13(0.15–8.40)	0.90	1.99(0.96–4.13)	0.06	2.09(1.19–3.68)	0.01
HoR-/HER2-	-	-	1.32(0.78–2.23)	0.31	1.47(0.97–2.23)	0.07
**AGE (year)**						
< 60	reference		reference		reference	
≥ 60	6.42(3.13–13.17)	< 0.0001	2.70(1.74–4.20)	< 0.0001	2.44(1.62–3.67)	< 0.0001

Abbreviations: *HR* hazard ratio, *HoR*: hormone receptor, *HER2* human epidermal growth factor receptor‐2

**Table 9:** Multivariate Cox regression analyses of overall survival for four molecular subtypes of T1c breast cancer patients in the matched cohort

**Variable**	HoR + /HER2-		HoR + /HER2 +		HoR-/HER2 +		HoR-/HER2-	
	Multivariate Analysis		Multivariate Analysis		Multivariate Analysis		Multivariate Analysis	
	HR (95%CI)	P-value	HR (95%CI)	*P*-value	HR (95%CI)	*P*-value	HR (95%CI)	*P*-value
**GRADE**								
I	reference		reference		reference		reference	
II	1.27(0.92–1.77)	0.14	1.02(0.54–1.93)	0.95	0.19(0.04–0.88)	0.03	1.71(0.69–4.24)	0.25
III	1.74(1.26–2.40)	< 0.01	1.12(0.58–2.13)	0.74	0.19(0.04–0.83)	0.03	2.19(0.90–5.31)	0.08
**SURGERY**								
Breast-conserving	reference		reference		reference		reference	
Total mastectomy	0.54(0.40–0.74)	< 0.0001	0.60(0.40–0.89)	0.01	0.41(0.21–0.78)	0.01	0.67(0.49–0.91)	0.01
Modified radical mastectomy	0.75(0.52–1.09)	0.13	0.52(0.29–0.93)	0.03	0.53(0.23–1.21)	0.13	0.55(0.36–0.85)	0.01
**RADIATION**								
No	reference		reference		reference		reference	
Yes	0.36(0.26–0.48)	< 0.0001	0.37(0.25–0.56)	< 0.0001	0.36(0.16–0.77)	0.01	0.48(0.35–0.66)	< 0.0001
**CHEMOTHERAPY**								
No	reference		reference		reference		reference	
Yes	0.61(0.51–0.74)	< 0.0001	0.42(0.30–0.58)	< 0.0001	0.33(0.19–0.59)	< 0.0001	0.35(0.27–0.44)	< 0.0001
**AGE (year)**								
< 60	reference		reference		reference		reference	
≥ 60	3.62(2.98–4.41)	< 0.0001	3.27(2.13–5.02)	< 0.0001	2.59(1.34–5.04)	< 0.01	2.01(1.50–2.69)	< 0.0001

Abbreviations: *HoR* hormone receptor, *HER2* human epidermal growth factor receptor‐2, *HR* hazard ratio

**Table 10:** Multivariate Cox regression analyses of overall survival for tumor grades of T1c breast cancer patients in the matched cohort

**Variable**	T1c: GRADEI		T1c: GRADEII		T1c: GRADEIII	
	**Multivariate Analysis**		**Multivariate Analysis**		**Multivariate Analysis**	
	HR (95%CI)	*P*-value	HR (95%CI)	*P*-value	HR (95%CI)	*P*-value
**SURGERY**						
Breast-conserving	reference		reference		reference	
Total mastectomy	0.63(0.26–1.52)	0.31	0.51(0.37–0.71)	< 0.0001	0.63(0.49–0.80)	< 0.01
Modified radical mastectomy	1.04(0.39–2.78)	0.94	0.60(0.39–0.93)	0.02	0.57(0.42–0.79)	< 0.01
**RADIATION**						
No	reference		reference		reference	
Yes	0.43(0.18–1.01)	0.05	0.40(0.29–0.55)	< 0.0001	0.43(0.34–0.55)	< 0.0001
**CHEMOTHERAPY**						
No	reference		reference		reference	
Yes	0.78(0.48–1.28)	0.33	0.53(0.43–0.66)	< 0.0001	0.44(0.37–0.53)	< 0.0001
**SUBTYPE**						
HoR + /HER2-	reference		reference		reference	
HoR + /HER2 +	1.70(0.92–3.14)	0.09	1.53(1.17–2.01)	< 0.01	1.26(0.96–1.65)	0.09
HoR-/HER2 +	6.54(0.88–48.72)	0.07	2.16(1.30–3.59)	< 0.01	1.14(0.79–1.63)	0.49
HoR-/HER2-	1.58(0.61–4.07)	0.34	2.05(1.53–2.73)	< 0.0001	1.81(1.50–2.20)	< 0.0001
**AGE (year)**						
< 60	reference		reference		reference	
≥ 60	3.38(1.98–5.77)	< 0.0001	3.68(2.82–4.81)	< 0.0001	2.19(1.81–2.66)	< 0.0001

Abbreviations: *HR* hazard ratio, *HoR* hormone receptor, *HER2* human epidermal growth factor receptor‐2

### External validation results

**Fig. 1:**
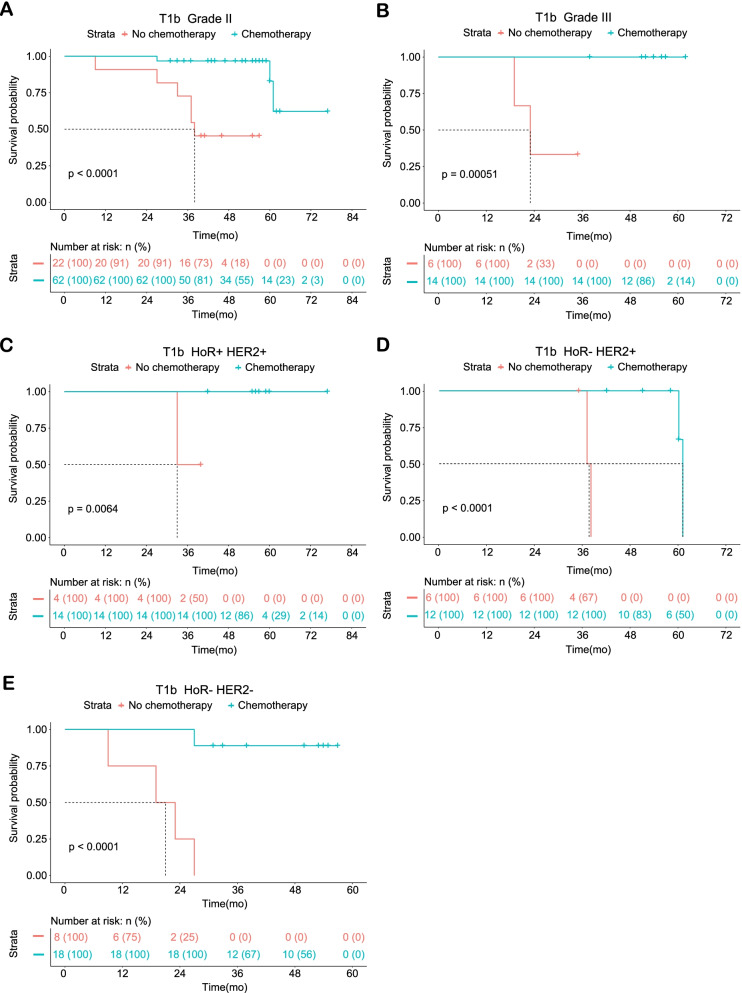
Statistically significant Kaplan–Meier survival curves of the adjuvant chemotherapy and no adjuvant chemotherapy groups according to the grades and molecular subtypes of T1bN0M0 breast cancer patients treated at the Northern Jiangsu People’s Hospital. **A** T1b Grade II; **B**: T1b Grade III; **C** T1b HoR + HER2 + ; **D** T1b HoR- HER2 + ; **E** T1b HoR- HER2-. Abbreviations: HoR, hormone receptor; HER2, human epidermal growth factor receptor 2

**Fig. 2:**
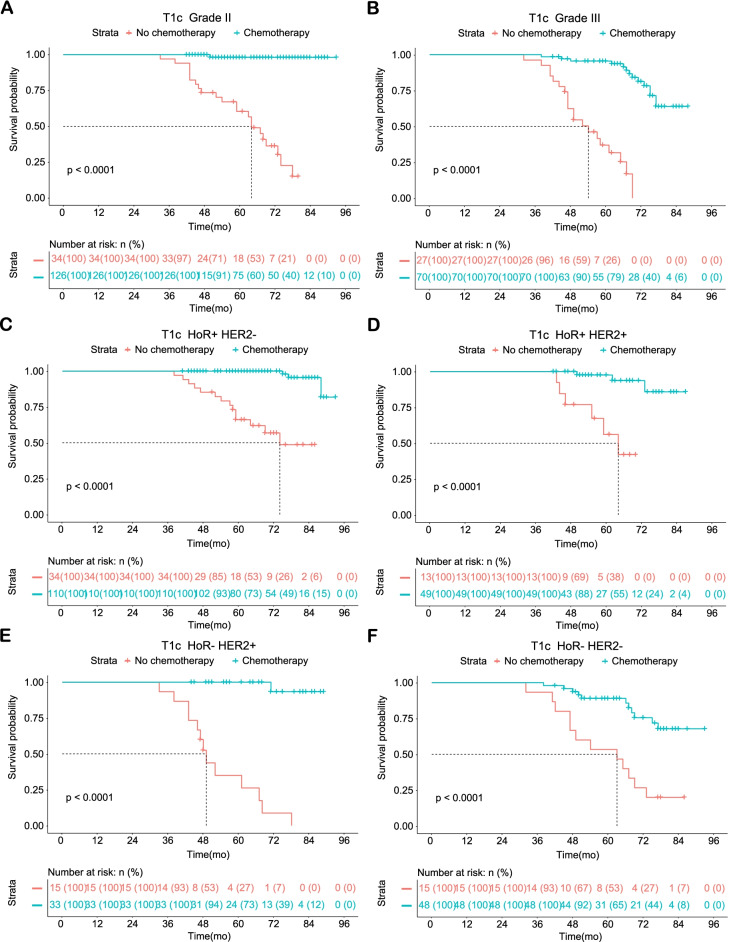
Statistically significant Kaplan–Meier survival curves of the adjuvant chemotherapy and no adjuvant chemotherapy groups according to the grades and molecular subtypes of T1cN0M0 breast cancer patients treated at the Northern Jiangsu People’s Hospital. **A** T1c Grade II; **B** T1c Grade III; **C** T1c HoR + HER2-; **D** T1c HoR + HER2 + ; **E** T1c HoR- HER2 + ; **F** T1c HoR- HER2-. Abbreviations: HoR, hormone receptor; HER2, human epidermal growth factor receptor 2

The median follow-up time was 60 months, as calculated by the reverse Kaplan–Meier method. The external validation cohort consisted of 545 early-stage BC patients and was comprised of three groups: T1a (*n* = 98), T1b (*n* = 130), and T1c (*n* = 317) (Table S22). Larger tumors tended to have worse differentiated tumor grades and often received adjuvant chemotherapy (*P* < 0.01). Results of the univariate and multivariate Cox regression analyses of T1a, T1b, and T1c are presented in Tables S23, S24, and S25. T1a early-stage BC patients did not benefit from adjuvant chemotherapy (*P* = 0.47). Nevertheless, patients with T1b (HR, 0.02; 95% CI, 0.00–0.09; *P* < 0.0001) and T1c (HR, 0.06; 95% CI, 0.03–0.11; *P* < 0.0001) BC had longer survival with adjuvant therapy. Kaplan–Meier survival curves were plotted for tumor grades and molecular subtypes: T1a (Figure S1), T1b (Figure S2), and T1c (Figure S3). All statistically significant results are summarized in Figs. 1 and 2. For T1a BC patients, adjuvant chemotherapy was ineffective for those with grade I (*P* = 0.43), grade II (*p* = 0.25), HoR + /HER2- (*P* = 0.75), HoR + /HER2 + (*P* = 0.26), and HoR-/HER2 + (*P* = 1). Grade III and triple-negative BC groups could not be plotted due to limited data available for T1a. For patients with grade II (*P* < 0.0001), grade III (*P* < 0.01), HoR + /HER2 + (*P* < 0.01), HoR-/HER2 + (*P* < 0.0001), and HoR/HER2- (*P* < 0.0001) T1b BC (Fig. 1A, B, C, D and E), adjuvant chemotherapy had beneficial effects on OS. Adjuvant chemotherapy improved OS (*P* < 0.0001) for patients with T1c BC (Fig. 2A, B, C, D, E, and F), but not for those with grade I (*P* = 0.07).

## Discussion

In spite of the dramatic increase in the number of early-stage BC patients [3–5, 16], the role of adjuvant chemotherapy in T1N0M0 BC remains controversial. Therefore, it is imperative to establish a safe, specific, and effective adjuvant chemotherapy strategy to guide treatment and improve the prognosis of these patients. In addition to creating the strategy, we utilized PSM and external validation dataset to verify the association between adjuvant chemotherapy and OS in T1N0M0 BC patients.

Adjuvant chemotherapy is recognized as a primary systematic adjuvant modality. However, it negatively influences survival and reduces the quality of life due to its short-term toxicities, including alopecia, nausea, vomiting, and fatigue, and potential long-term side-effects, including myelosuppression, cardiovascular toxicity, neurotoxicity, marrow neoplasm, and cessation of menses and fertility [17–20]. Early-stage BC patients are expected to survive their cancer diagnosis. As adjuvant chemotherapy associated toxicity could cause death, it is better to consider OS instead of BC-specific mortality as an end-point [13, 21]. Furthermore, our study suggests that adjuvant chemotherapy possibly accelerates death for some HoR + /HER2- T1aN0M0, and T1bN0M0 patients.

Postmastectomy radiation therapy is widely considered to reduce the risk of local recurrence and mortality, especially in patients with locally advanced tumors, as these patients are at a high risk due to large tumors and axillary lymph node involvement [22–24]. However, the majority of T1N0M0 BC patients prefer to undergo breast-conserving surgery instead of mastectomy. Adjuvant radiotherapy is a locoregional treatment that is often combined with breast-conserving surgery to achieve local control benefits and OS advantages [25–30]. These results are consistent with the results from our study.

The results from the SEER database were in contradiction to the results obtained from the external validation cohort. The external validation results indicated that patients with grade II T1bN0M0 could acquire survival benefit from adjuvant chemotherapy. However, this was not observed in the results from the SEER database. There are two explanations for this phenomenon. Firstly, the data used for external validation were relatively limited, Therefore, inevitable deviations might have occurred during the statistical analyses. Secondly, the two cohorts of data were derived from China and the United States, respectively. Admittedly, the factors that affect a patient’s lifetime vary from country to country and are influenced by cultural barriers, ethnic differences, and genetics [31]. Consequently, the final conclusions refer to the results obtained from the SEER database.

The results from our study, the guidelines of the National Comprehensive Cancer Network (NCCN), and the guidelines of the St. Gallen International BC Conference (BCC) are nearly identical [32, 33]. The NCCN suggests the following:

1. For node-negative HoR + /HER2- BC, if the tumor is 0.5 cm or smaller, adjuvant chemotherapy is not recommended. If the tumor is larger than 0.5 cm, then performing a 21-gene reverse-transcription polymerase chain reaction assay (Oncotype DX) is strongly recommended [34–36]. 
  a If the recurrence score is ≥ 31, the risk of recurrence is high and adjuvant chemotherapy is recommended.
  b If the recurrence score is between 26–30, the risk of recurrence is moderate and the decision to perform adjuvant chemotherapy is based on other clinical factors.
  c If the recurrence score is < 26, the risk of recurrence is low and adjuvant chemotherapy is not recommended.
2. For node-negative HoR + /HER2 + BC, if the tumor is 1.0 cm or smaller, it is unclear whether adjuvant chemotherapy is required. However, adjuvant chemotherapy is recommended for T1a category 2B, which means that there is an NCCN consensus that intervention is appropriate based on lower-level evidence. If the tumor is larger than 1.0 cm, adjuvant chemotherapy is recommended.
3. For node-negative HoR-/HER2 + BC, adjuvant chemotherapy is recommended. Also, adjuvant chemotherapy is recommended for category 2B when the tumor is smaller than 0.5 cm.
4. For node-negative HoR-/HER2- BC, if the tumor is smaller than 0.5 cm, adjuvant chemotherapy is not recommended. However, adjuvant chemotherapy is necessary for all other cases.

The BCC guidelines are different but somewhat similar to the NCCN guidelines [33]. Routine adjuvant chemotherapy is not recommended for T1aN0M0 BC; this is similar to the results from our study. The BCC panel recommends adjuvant chemotherapy for HER2 + and triple-negative BC (TNBC) stage T1bN0M0 and higher. For ER + /HER2- T1N0M0 BC, regardless of luminal-A-like qualities (strongly ER + and PR + , HER2-, with lower grade and proliferation markers) or luminal-B-like tumors, the BCC panel does not recommend adjuvant chemotherapy for patients with low genomic risk scores, according to the Oncotype DX and 70-gene signature tests (MammaPrint) [37–40]. Additionally, the European Society for Medical Oncology guidelines are in agreement with the St. Gallen guidelines regarding adjuvant chemotherapy for early-stage BC [41].

In our opinion, which is also supported by the St. Gallen guidelines, adjuvant chemotherapy should not be performed for T1aN0M0 BC patients. For HoR + /HER2- T1bN0M0 and T1cN0M0 BC, adjuvant chemotherapy is recommended for grade II and grade III T1cN0M0 BC when no genetic signature test has been performed or when the 21-gene assay indicates a medium risk. If the conditions are suitable, we propose to perform genetic testing for these patients, in accordance with the guidelines. For the other three molecular subtypes, adjuvant chemotherapy is recommended for stage T1bN0M0 and higher; this is also mentioned in the St. Gallen guidelines. Previous retrospective studies have demonstrated the survival benefits of adjuvant chemotherapy for patients with T1cN0M0 TNBC [42–44]. We incorporated tumor grade, which is an independent prognostic indicator, to assess the effects of adjuvant chemotherapy [45–47]. Patients, including those with TNBC, can be exempt from adjuvant chemotherapy if they have grade I/II T1bN0M0 and grade I T1cN0M0 BC [48].

Our study has several limitations. Firstly, the SEER database lacked information regarding the genetic background of the patients such as the 21-gene assay and schemes, and data regarding the therapies given to the patients, including details about the dosages of adjuvant chemotherapy and endocrine therapies. Secondly, this study lacked data regarding the muscle mass of the patients. Previous studies reported that chemotherapy could increase the hematological toxicity of BC patients with a low muscle mass, which might further affect their OS [49, 50]. Thirdly, because the endpoint was OS, age was a significant factor that could not be included to evaluate the effects of adjuvant chemotherapy. Fourthly, because we used a retrospective cohort population, inevitable selection bias might have affected the conclusions. Further large-scale, prospective, randomized, controlled clinical trials are warranted to accurately identify the outcomes.

## Conclusions

Our study found that adjuvant chemotherapy is not beneficial and might even be detrimental to T1aN0M0 BC patients. Moreover, adjuvant chemotherapy is recommended for patients with tumor grade III T1bN0M0 and grade II/III T1cN0M0 BC, but not to patients with HoR + /HER2- BC. Regarding the molecular subtype HoR + /HER2-, in the absence of genetic testing, adjuvant chemotherapy is recommended for tumor grade II and grade III T1cN0M0 BC. However, further randomized, controlled clinical trials are needed to confirm these results.

## Supplementary Information

**Additional file 1: Figure S1.** Kaplan–Meier survival curves of the chemotherapy and nochemotherapy groups according to grades and molecular subtypes of T1a breastcancer patients treated at Northern Jiangsu People’s Hospital. (A) Grade I; (B)grade II; (C) HoR+/HER2-; (D) HoR+/HER2+; and (E) HoR-/HER2+. Abbreviations: HoR: hormonereceptor; HER2: human epidermal growth factor receptor‐2.

**Additional file 2: Figure S2.** Kaplan–Meier survival curves of the chemotherapy and nochemotherapy groups according to the grades and molecular subtypes of T1bbreast cancer patients treated at Northern Jiangsu People’s Hospital. (A) GradeI; (B) grade II; (C) grade III; (D) HoR+/HER2-; (E) HoR+/HER2+; (F) HoR-/HER2+;and (G) HoR-/HER2-. Abbreviations: HoR: hormone receptor; HER2: human epidermal growthfactor receptor‐2.

**Additional file 3: Figure S3.** Kaplan–Meier survival curves of the chemotherapy and nochemotherapy groups according to the grades and molecular subtypes of T1cbreast cancer patients treated at Northern Jiangsu People’s Hospital. (A) GradeI; (B): grade II; (C) grade III; (D) HoR+/HER2-; (E) HoR+/HER2+; (F) HoR-/HER2+;and (G) HoR-/HER2-. Abbreviations: HoR: hormone receptor; HER2: human epidermal growthfactor receptor‐2.

**Additional file 4: Table S1.** Demographic andclinical characteristics of the included T1N0M0 breast cancer patients in theSEER database.

**Additional file 5: Table S2.** Multivariate Cox regressionanalyses of overall survival for T1, T1a, T1b, and T1c breast cancer patients.

**Additional file 6: Table S3.** Multivariate Cox regressionanalyses of overall survival for T1, T1a, T1b, and T1c breast cancer patientsregarding treatment methods.

**Additionalfile 7: Table S4.** MultivariableCox regression analyses of overall survival forfour molecular subtypes in T1a breast cancer patients.

**Additional file 8: Table S5.** Multivariable Cox regression analyses of overall survival for tumor grades in T1a breast cancer patients.

**Additional file 9: Table S6.** Multivariable Cox regression analyses of overall survival for molecularsubtypes in T1b breast cancer patients.

**Additional file 10: Table S7.**Multivariable Cox regression analyses of overall survival for tumorgrades in T1b breast cancer patients.

**Additional file 11: Table S8. **Multivariable Coxregression analyses of overall survival for molecular subtypes in T1c breast cancer patients.

**Additional file 12: Table S9. **Multivariable Coxregression analyses of overall survival for tumor grades in T1c breast cancerpatients.

**Additional file 13: Table S10. **Multivariable Coxregression analyses of overall survival for tumor grades in HoR+/HER2- T1abreast cancer patients.

**Additional file 14: Table S11. **Multivariable Cox regression analyses of overall survival for tumorgrades in HoR+/HER2+ T1abreast cancer patients.

**Additional file 15: Table S12. **Multivariable Cox regression analyses of overall survival for tumorgrades in HoR-/HER2+ T1a breast cancer patients.

**Additional file 16: Table S13. **Multivariable Cox regression analyses of overall survival for tumorgrades in HoR-/HER2- T1a breast cancer patients.

**Additional file 17: Table S14. **Multivariable Coxregression analyses of overall survival for tumor grades in HoR+/HER2- T1b breastcancer patients.

**Additional file 18: Table S15. **Multivariable Coxregression analyses of overall survival for tumor grades in HoR+/HER2+ T1b breast cancer patients.

**Additional file 19: Table S16. **Multivariable Coxregression analyses of overall survival for tumor grades in HoR-/HER2+ T1b breast cancer patients.

**Additional file 20: Table S17. **MultivariableCox regression analysesof overall survival for tumor grades in HoR-/HER2- T1b breast cancer patients.

**Additional file 21: Table S18. **Multivariable Cox regression analyses of overall survival for tumorgrades in HoR+/HER2- T1c breast cancer patients.

**Additional file 22: Table S19. **Multivariable Cox regression analyses of overall survival fortumor grades in HoR+/HER2+ T1c breast cancer patients.

**Additional file 23: Table S20. **Multivariable Cox regression analyses of overall survival for tumorgrades in HoR-/HER2+ T1c breast cancer patients.

**Additional file 24: Table S21. **Multivariable Cox regression analysesof overall survival for tumor grades in HoR-/HER2- T1c breast cancer patients.

**Additional file 25: Table S22. **Demographic and clinicalcharacteristics of the included T1N0M0 breast cancer patients in Northern Jiangsu People’sHospital.

**Additional file 26: Table S23. **Univariableand multivariable Cox regression analyses of overallsurvival for T1a breast cancer patients in Northern Jiangsu People’s Hospital.

**Additional file 27: Table S24. **Univariable and multivariable Cox regression analyses ofoverall survival for T1b breast cancer patients in Northern Jiangsu People’s Hospital.

**Additional file 28: Table S25. **Univariable and multivariable Cox regression analyses ofoverall survival for T1c breast cancer patients in Northern Jiangsu People’sHospital.

## Data Availability

The data that support the findings of this study are available from the corresponding authors upon reasonable request. The dataset from SEER database generated and/or analyzed during the current study are available in the SEER dataset repository (https://seer.cancer.gov/data/).

## Abbreviations

BC: Breast cancer
SEER: Surveillance, Epidemiology, and End Results
PSM: Propensity score matching
OS: Overall survival
HoR: Hormone receptor
ER: Estrogen receptor
PR: Progesterone receptor
HER‐2: Human epidermal growth factor receptor‐2
HR: Hazard ratio
NCCN: National Comprehensive Cancer Network
BCC: Breast Cancer Conference
TNBC: Triple-negative breast cancer
