# Supplementary material for: Impact of adjuvant chemotherapy on T1N0M0 breast cancer patients: a propensity score matching study based on SEER database and external cohort
Source: BMC Cancer. 2022 Aug 8;22:863. doi: 10.1186/s12885-022-09952-z (PMC9358893; doi:10.1186/s12885-022-09952-z)
Supplement: Supplementary file 1 — Additional file 1: Figure S1. Kaplan–Meier survival curves of the chemotherapy and nochemotherapy groups according to grades and molecular subtypes of T1a breastcancer patients treated at Northern Jiangsu People’s Hospital. (A) Grade I; (B)grade II; (C) HoR+/HER2-; (D) HoR+/HER2+; and (E) HoR-/HER2+. Abbreviations: HoR: hormonereceptor; HER2: human epidermal growth factor receptor‐2. [file 12885_2022_9952_MOESM1_ESM.docx]

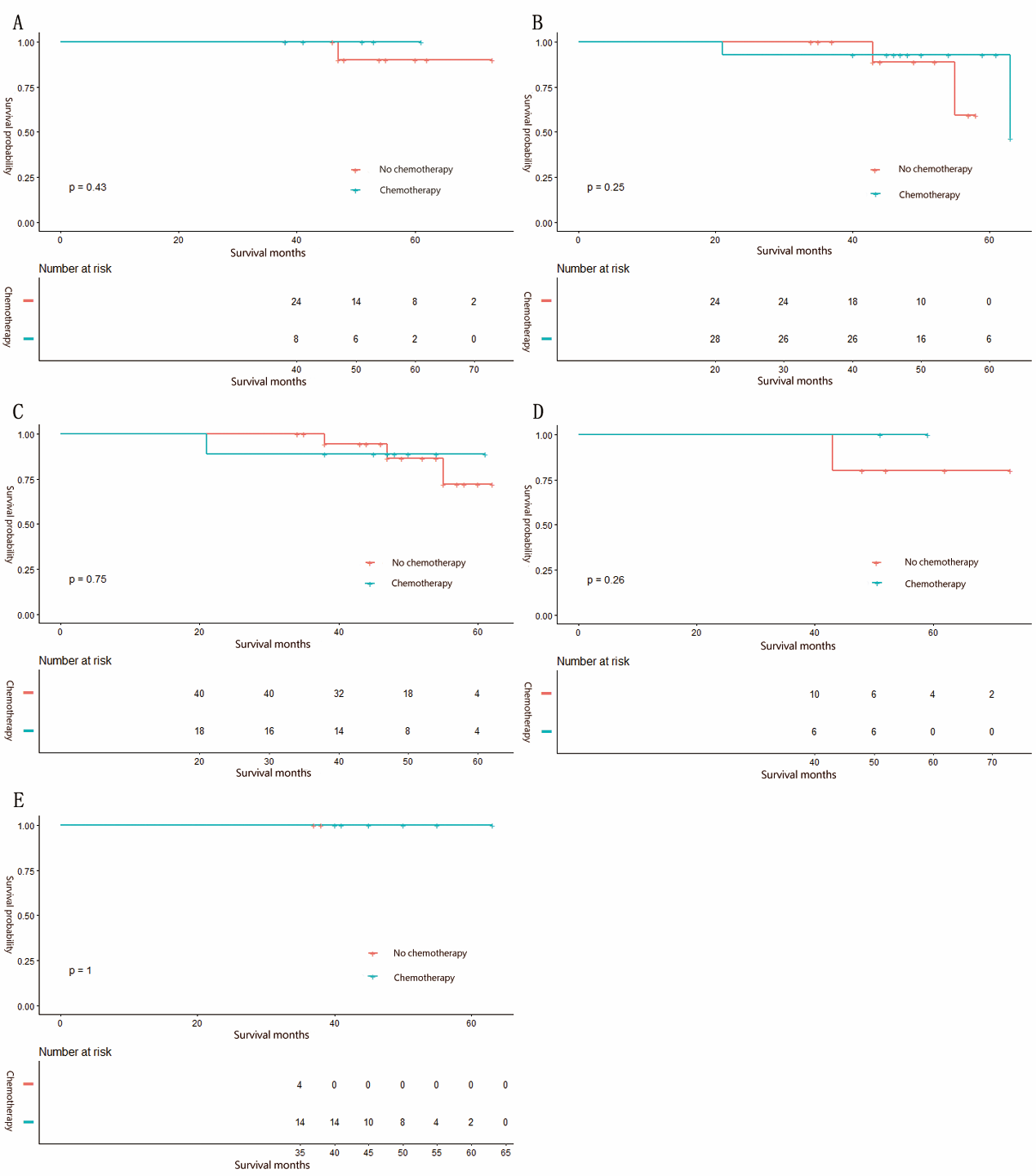
Figure S1: Kaplan–Meier survival curves of the chemotherapy and no chemotherapy groups according to grades and molecular subtypes of T1a breast cancer patients treated at Northern Jiangsu People’s Hospital. (A) Grade I; (B) grade II; (C) HoR+/HER2-; (D) HoR+/HER2+; and (E) HoR-/HER2+. Abbreviations: HoR: hormone receptor; HER2: human epidermal growth factor receptor‐2
