# Supplementary material for: Impact of adjuvant chemotherapy on T1N0M0 breast cancer patients: a propensity score matching study based on SEER database and external cohort
Source: BMC Cancer. 2022 Aug 8;22:863. doi: 10.1186/s12885-022-09952-z (PMC9358893; doi:10.1186/s12885-022-09952-z)
Supplement: Supplementary file 2 — Additional file 2: Figure S2. Kaplan–Meier survival curves of the chemotherapy and nochemotherapy groups according to the grades and molecular subtypes of T1bbreast cancer patients treated at Northern Jiangsu People’s Hospital. (A) GradeI; (B) grade II; (C) grade III; (D) HoR+/HER2-; (E) HoR+/HER2+; (F) HoR-/HER2+;and (G) HoR-/HER2-. Abbreviations: HoR: hormone receptor; HER2: human epidermal growthfactor receptor‐2. [file 12885_2022_9952_MOESM2_ESM.docx]

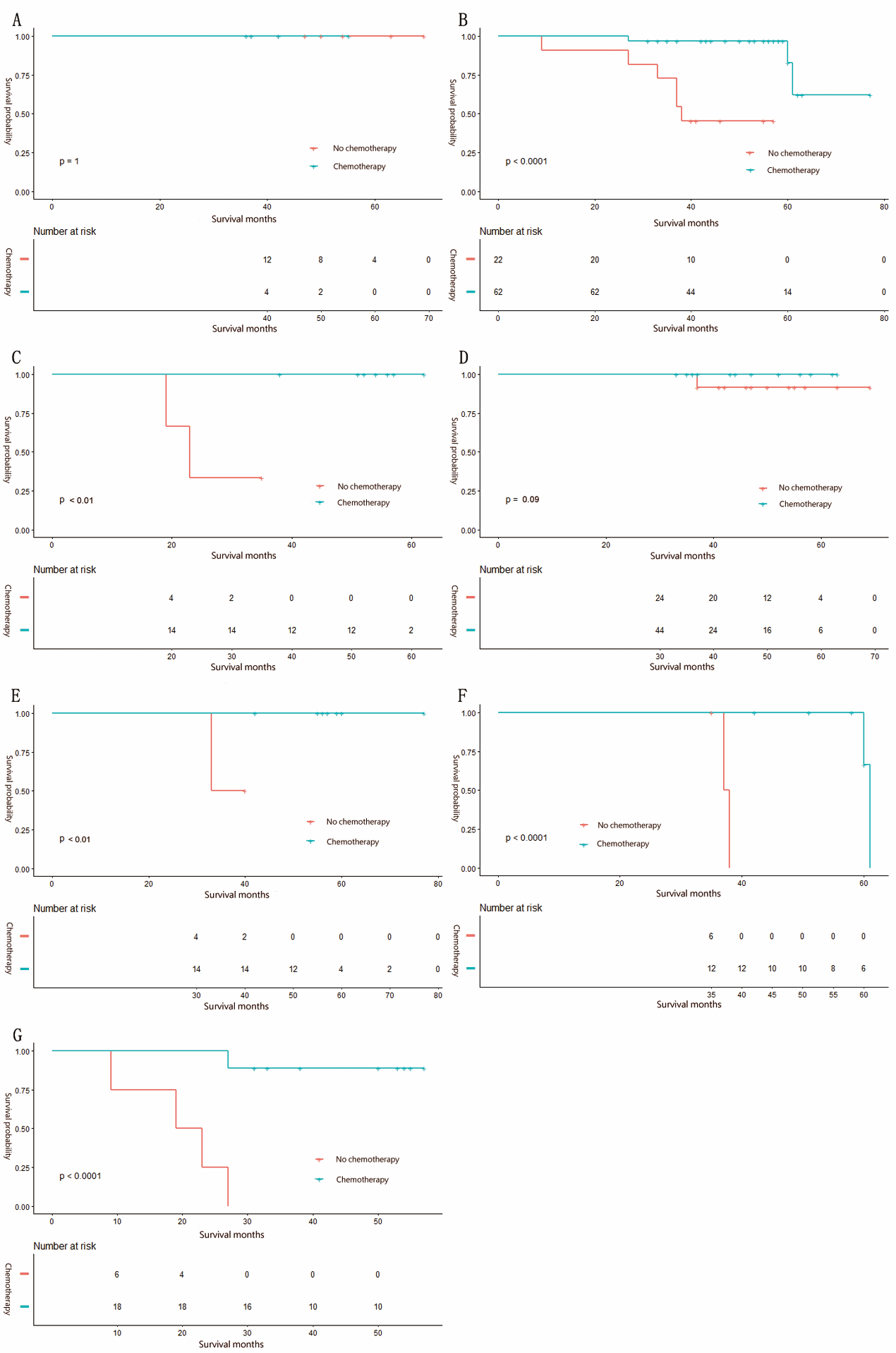
Figure S2: Kaplan–Meier survival curves of the chemotherapy and no chemotherapy groups according to the grades and molecular subtypes of T1b breast cancer patients treated at Northern Jiangsu People’s Hospital. (A) Grade I; (B) grade II; (C) grade III; (D) HoR+/HER2-; (E) HoR+/HER2+; (F) HoR-/HER2+; and (G) HoR-/HER2-. Abbreviations: HoR: hormone receptor; HER2: human epidermal growth factor receptor‐2
