# Supplementary material for: Impact of adjuvant chemotherapy on T1N0M0 breast cancer patients: a propensity score matching study based on SEER database and external cohort
Source: BMC Cancer. 2022 Aug 8;22:863. doi: 10.1186/s12885-022-09952-z (PMC9358893; doi:10.1186/s12885-022-09952-z)
Supplement: Supplementary file 4 — Additional file 4: Table S1. Demographic andclinical characteristics of the included T1N0M0 breast cancer patients in theSEER database. [file 12885_2022_9952_MOESM4_ESM.docx]

Table S1: Demographic and clinical characteristics of the included T1N0M0 breast cancer patients in the SEER database.

| Demographic and Clinical Characteristic | T1 | T1a | T1b | T1c | P-value |
| --- | --- | --- | --- | --- | --- |
|  | N=75139 | N=10073 | N=24951 | N=40115 |  |
| **GRADE** |  |  |  |  | p<0.01 |
| I | 25122 (33.43%) | 4382 (43.50%) | 10256 (41.10%) | 10484 (26.13%) |  |
| II | 34469 (45.87%) | 4274 (42.43%) | 10897 (43.67%) | 19298 (48.11%) |  |
| III | 15548 (20.69%) | 1417 (14.07%) | 3798 (15.22%) | 10333 (25.76%) |  |
| **SURGERY** |  |  |  |  | p<0.01 |
| Breast-conserving | 50020 (66.57%) | 6454 (64.07%) | 17688 (70.89%) | 25878 (64.51%) |  |
| Total mastectomy | 19737 (26.27%) | 2956 (29.35%) | 5762 (23.09%) | 11019 (27.47%) |  |
| Modified radical mastectomy | 5382 (7.16%) | 663 (6.58%) | 1501 (6.02%) | 3218 (8.02%) |  |
| **RADIATION** |  |  |  |  | p<0.01 |
| No | 31604 (42.06%) | 4482 (44.50%) | 9566 (38.34%) | 17556 (43.76%) |  |
| Yes | 43535 (57.94%) | 5591 (55.50%) | 15385 (61.66%) | 22559 (56.24%) |  |
| **CHEMOTHERAPY** |  |  |  |  | p<0.01 |
| No | 59890 (79.71%) | 9225 (91.58%) | 21303 (85.38%) | 29362 (73.19%) |  |
| Yes | 15249 (20.29%) | 848 (8.42%) | 3648 (14.62%) | 10753 (26.81%) |  |
| **SUBTYPE** |  |  |  |  | p<0.01 |
| HoR+/HER2- | 61196 (81.44%) | 8077 (80.18%) | 21211 (85.01%) | 31908 (79.54%) |  |
| HoR+/HER2+ | 5745 (7.65%) | 860 (8.54%) | 1588 (6.36%) | 3297 (8.22%) |  |
| HoR-/HER2+ | 1951 (2.60%) | 440 (4.37%) | 486 (1.95%) | 1025 (2.56%) |  |
| HoR-/HER2- | 6247 (8.31%) | 696 (6.91%) | 1666 (6.68%) | 3885 (9.68%) |  |
| **AGE (year)** |  |  |  |  | p<0.01 |
| ＜60 | 31387 (41.77%) | 4393 (43.61%) | 9753 (39.09%) | 17241 (42.98%) |  |
| ≥60 | 43752 (58.23%) | 5680 (56.39%) | 15198 (60.91%) | 22874 (57.02%) |  |

Abbreviations: HoR: hormone receptor; HER‐2: human epidermal growth factor receptor‐2
