# Supplementary material for: Impact of adjuvant chemotherapy on T1N0M0 breast cancer patients: a propensity score matching study based on SEER database and external cohort
Source: BMC Cancer. 2022 Aug 8;22:863. doi: 10.1186/s12885-022-09952-z (PMC9358893; doi:10.1186/s12885-022-09952-z)
Supplement: Supplementary file 5 — Additional file 5: Table S2. Multivariate Cox regressionanalyses of overall survival for T1, T1a, T1b, and T1c breast cancer patients. [file 12885_2022_9952_MOESM5_ESM.docx]

Table S2: Multivariate Cox regression analyses of overall survival for T1, T1a, T1b, and T1c breast cancer patients.

| Variables | T1 | | T1a | | T1b | | T1c | |
| --- | --- | --- | --- | --- | --- | --- | --- | --- |
|  | Multivariate Analysis | | Multivariate Analysis | | Multivariate Analysis | | Multivariate Analysis | |
|  | HR (95%CI) | P-value | HR (95%CI) | P-value | HR (95%CI) | P-value | HR (95%CI) | P-value |
| **GRADE** |  |  |  |  |  |  |  |  |
| I | reference |  | reference |  | reference |  | reference |  |
| II | 1.06(0.99-1.14) | 0.10 | 1.08(0.87-1.33) | 0.50 | 1.01(0.89-1.14) | 0.92 | 1.10(1.00-1.21) | 0.04 |
| III | 1.37(1.25-1.50) | <0.0001 | 1.13(0.81-1.58) | 0.46 | 1.19(0.99-1.43) | 0.06 | 1.48(1.32-1.66) | <0.0001 |
| **SURGERY** |  |  |  |  |  |  |  |  |
| Breast-conserving | reference |  | reference |  | reference |  | reference |  |
| Total mastectomy | 0.59(0.55-0.65) | <0.0001 | 0.66(0.50-0.87) | <0.01 | 0.74(0.63-0.88) | <0.01 | 0.54(0.48-0.60) | <0.0001 |
| Modified radical mastectomy | 0.69(0.61-0.77) | <0.0001 | 0.71(0.48-1.03) | 0.08 | 0.96(0.77-1.19) | 0.70 | 0.60(0.52-0.69) | <0.0001 |
| **RADIATION** |  |  |  |  |  |  |  |  |
| No | reference |  | reference |  | reference |  | reference |  |
| Yes | 0.35(0.32-0.38) | <0.0001 | 0.35(0.27-0.45) | <0.0001 | 0.44(0.38-0.51) | <0.0001 | 0.32(0.29-0.35) | <0.0001 |
| **CHEMOTHERAPY** |  |  |  |  |  |  |  |  |
| No | reference |  | reference |  | reference |  | reference |  |
| Yes | 0.61(0.55-0.67) | <0.0001 | 1.23(0.85-1.78) | 0.27 | 0.72(0.59-0.88) | <0.0001 | 0.54(0.48-0.60) | <0.0001 |
| **SUBTYPE** |  |  |  |  |  |  |  |  |
| HoR+/HER2- | reference |  | reference |  | reference |  | reference |  |
| HoR+/HER2+ | 1.25(1.11-1.41) | <0.01 | 0.67(0.43-1.05) | 0.08 | 1.32(1.05-1.68) | 0.02 | 1.33(1.15-1.54) | <0.0001 |
| HoR-/HER2+ | 1.47(1.23-1.75) | <0.0001 | 1.44(0.95-2.21) | 0.09 | 1.64(1.14-2.37) | 0.01 | 1.36(1.08-1.73) | 0.01 |
| HoR-/HER2- | 1.82(1.65-2.01) | <0.0001 | 1.20(0.83-1.74) | 0.33 | 1.65(1.33-2.05) | <0.0001 | 1.99(1.76-2.25) | <0.0001 |
| **AGE (year)** |  |  |  |  |  |  |  |  |
| ＜60 | reference |  | reference |  | reference |  | reference |  |
| ≥60 | 3.45(3.19-3.74) | <0.0001 | 3.43(2.67-4.41) | <0.0001 | 3.72(3.17-4.36) | <0.0001 | 3.35(3.03-3.70) | <0.0001 |
| **T1** |  |  |  |  |  |  |  |  |
| T1a | reference |  |  |  |  |  |  |  |
| T1b | 1.23(1.10-1.37) | <0.01 |  |  |  |  |  |  |
| T1c | 1.80(1.62-1.99) | <0.0001 |  |  |  |  |  |  |

Abbreviations: HR: hazard ratio; HoR: hormone receptor; HER‐2: human epidermal growth factor receptor‐2
