# Supplementary material for: Impact of adjuvant chemotherapy on T1N0M0 breast cancer patients: a propensity score matching study based on SEER database and external cohort
Source: BMC Cancer. 2022 Aug 8;22:863. doi: 10.1186/s12885-022-09952-z (PMC9358893; doi:10.1186/s12885-022-09952-z)
Supplement: Supplementary file 6 — Additional file 6: Table S3. Multivariate Cox regressionanalyses of overall survival for T1, T1a, T1b, and T1c breast cancer patientsregarding treatment methods. [file 12885_2022_9952_MOESM6_ESM.docx]

Table S3: Multivariate Cox regression analyses of overall survival for T1, T1a, T1b, and T1c breast cancer patients regarding treatment methods.

| Variables | T1 | | T1a | | T1b | | T1c | |
| --- | --- | --- | --- | --- | --- | --- | --- | --- |
|  | Multivariate Analysis | | Multivariate Analysis | | Multivariate Analysis | | Multivariate Analysis | |
|  | HR (95%CI) | P-value | HR (95%CI) | P-value | HR (95%CI) | P-value | HR (95%CI) | P-value |
| **GRADE** |  |  |  |  |  |  |  |  |
| I | reference |  | reference |  | reference |  | reference |  |
| II | 1.07(0.99-1.14) | 0.08 | 1.07(0.87-1.32) | 0.53 | 1.02(0.90-1.16) | 0.71 | 1.10(1.00-1.21) | 0.04 |
| III | 1.38(1.26-1.51) | <0.0001 | 1.13(0.81-1.58) | 0.48 | 1.21(1.00-1.45) | 0.05 | 1.49(1.33-1.68) | <0.0001 |
| **TREATMENT METHODS** |  |  |  |  |  |  |  |  |
| BCS with RT | reference |  | reference |  | reference |  | reference |  |
| BCS | 3.07(2.84-3.32) | <0.0001 | 3.01(2.31-3.93) | <0.0001 | 2.53(2.17-2.94) | <0.0001 | 3.32(3.01-3.67) | <0.0001 |
| Total mastectomy | 1.71(1.59-1.83) | <0.0001 | 1.91(1.52-2.40) | <0.0001 | 1.70(1.48-1.95) | <0.0001 | 1.69(1.54-1.85) | <0.0001 |
| MRM | 1.91(1.72-2.12) | <0.0001 | 2.05(1.44-2.93) | <0.0001 | 2.02(1.64-2.48) | <0.0001 | 1.86(1.63-2.12) | <0.0001 |
| **CHEMOTHERAPY** |  |  |  |  |  |  |  |  |
| No | reference |  | reference |  | reference |  | reference |  |
| Yes | 0.58(0.53-0.64) | <0.0001 | 1.17(0.80-1.71) | 0.42 | 0.65(0.53-0.81) | <0.0001 | 0.52(0.47-0.59) | <0.0001 |
| **SUBTYPE** |  |  |  |  |  |  |  |  |
| HoR+/HER2- | reference |  | reference |  | reference |  | reference |  |
| HoR+/HER2+ | 1.25(1.11-1.41) | <0.01 | 0.62(0.39-1.00) | 0.05 | 1.37(1.08-1.74) | 0.01 | 1.33(1.14-1.53) | <0.0001 |
| HoR-/HER2+ | 1.48(1.23-1.77) | <0.0001 | 1.38(0.89-2.13) | 0.15 | 1.71(1.18-2.48) | <0.01 | 1.39(1.10-1.76) | <0.01 |
| HoR-/HER2- | 1.81(1.63-2.00) | <0.0001 | 1.22(0.85-1.77) | 0.29 | 1.63(1.30-2.03) | <0.0001 | 1.98(1.75-2.24) | <0.0001 |
| **AGE (year)** |  |  |  |  |  |  |  |  |
| ＜60 | reference |  | reference |  | reference |  | reference |  |
| ≥60 | 3.47(3.20-3.77) | <0.0001 | 3.41(2.64-4.39) | <0.0001 | 3.69(3.14-4.34) | <0.0001 | 3.39(3.01-3.67) | <0.0001 |

Abbreviations: HR: hazard ratio; BCS: breast-conserving surgery; RT: radiotherapy; MRM: modified radical mastectomy; HoR: hormone receptor; HER‐2: human epidermal growth factor receptor‐2
