# Supplementary material for: Impact of adjuvant chemotherapy on T1N0M0 breast cancer patients: a propensity score matching study based on SEER database and external cohort
Source: BMC Cancer. 2022 Aug 8;22:863. doi: 10.1186/s12885-022-09952-z (PMC9358893; doi:10.1186/s12885-022-09952-z)
Supplement: Supplementary file 7 — Additionalfile 7: Table S4. MultivariableCox regression analyses of overall survival forfour molecular subtypes in T1a breast cancer patients. [file 12885_2022_9952_MOESM7_ESM.docx]

Table S4: Multivariable Cox regression analyses of overall survival for four molecular subtypes in T1a breast cancer patients.

| **Variables** | T1a:HoR+/HER2- | | T1a:HoR+/HER2+ | | T1a:HoR-/HER2+ | | T1a:HoR-/HER2- | |
| --- | --- | --- | --- | --- | --- | --- | --- | --- |
|  | **Multivariate Analysis** | | **Multivariate Analysis** | | **Multivariate Analysis** | | **Multivariate Analysis** | |
|  | HR (95%CI) | P-value | HR (95%CI) | P-value | HR (95%CI) | P-value | HR (95%CI) | P-value |
| **GRADE** |  |  |  |  |  |  |  |  |
| I | reference |  | reference |  | reference |  | reference |  |
| II | 1.06(0.85-1.32) | 0.62 | 1.52(0.43-5.33) | 0.51 | 0.69(0.09-5.33) | 0.72 | 1.02(0.29-3.50) | 0.98 |
| III | 1.11(0.73-1.69) | 0.63 | 1.34(0.31-5.71) | 0.69 | 0.80(0.10-6.23) | 0.83 | 0.97(0.29-3.31) | 0.96 |
| **SURGERY** |  |  |  |  |  |  |  |  |
| Breast-conserving | reference |  | reference |  | reference |  | reference |  |
| Total mastectomy | 0.64(0.47-0.87) | <0.0001 | 1.27(0.29-5.54) | 0.75 | 2.94(0.59-14.57) | 0.19 | 0.40(0.18-0.91) | 0.03 |
| Modified radical mastectomy | 0.76(0.50-1.17) | 0.21 | 0.99(0.14-7.10) | 0.99 | 3.65(0.66-20.28) | 0.14 | 0.17(0.04-0.80) | 0.02 |
| **RADIATION** |  |  |  |  |  |  |  |  |
| No | reference |  | reference |  | reference |  | reference |  |
| Yes | 0.34(0.26-0.45) | <0.0001 | 0.68(0.15-2.97) | 0.60 | 2.30(0.50-10.69) | 0.29 | 0.18(0.08-0.43) | <0.0001 |
| **CHEMOTHERAPY** |  |  |  |  |  |  |  |  |
| No | reference |  | reference |  | reference |  | reference |  |
| Yes | 2.35(1.52-3.62) | 0.11 | 0.56(0.16-1.94) | 0.36 | 0.54(0.20-1.46) | 0.22 | 0.53(0.20-1.40) | 0.20 |
| **AGE (year)** |  |  |  |  |  |  |  |  |
| ＜60 | reference |  | reference |  | reference |  | reference |  |
| ≥60 | 3.88(2.88-5.24) | <0.0001 | 5.28(1.90-14.67) | <0.0001 | 2.35(1.02-5.39) | 0.04 | 1.43(0.71-2.88) | 0.32 |

Abbreviations: HR: hazard ratio; HoR: hormone receptor; HER‐2: human epidermal growth factor receptor‐2
