# Supplementary material for: Impact of adjuvant chemotherapy on T1N0M0 breast cancer patients: a propensity score matching study based on SEER database and external cohort
Source: BMC Cancer. 2022 Aug 8;22:863. doi: 10.1186/s12885-022-09952-z (PMC9358893; doi:10.1186/s12885-022-09952-z)
Supplement: Supplementary file 8 — Additional file 8: Table S5. Multivariable Cox regression analyses of overall survival for tumor grades in T1a breast cancer patients. [file 12885_2022_9952_MOESM8_ESM.docx]

Table S5: Multivariable Cox regression analyses of overall survival for tumor grades in T1a breast cancer patients.

| **Variable** | T1a：GRADEⅠ | | T1a：GRADEⅡ | | T1a：GRADEⅢ | |
| --- | --- | --- | --- | --- | --- | --- |
|  | **Multivariate Analysis** | | **Multivariate Analysis** | | **Multivariate Analysis** | |
|  | HR (95%CI) | P-value | HR (95%CI) | P-value | HR (95%CI) | P-value |
| **SURGERY** |  |  |  |  |  |  |
| Breast-conserving | reference |  | reference |  | reference |  |
| Total mastectomy | 0.75(0.49-1.15) | 0.18 | 0.55(0.36-0.82) | <0.01 | 0.74(0.34-1.61) | 0.44 |
| Modified radical mastectomy | 0.98(0.54-1.76) | 0.94 | 0.55(0.31-1.00) | 0.05 | 0.61(0.22-1.68) | 0.34 |
| **RADIATION** |  |  |  |  |  |  |
| No | reference |  | reference |  | reference |  |
| Yes | 0.36(0.25-0.54) | <0.0001 | 0.33(0.22-0.49) | <0.0001 | 0.33(0.15-0.72) | 0.01 |
| **CHEMOTHERAPY** |  |  |  |  |  |  |
| No | reference |  | reference |  | reference |  |
| Yes | 1.14(0.46-2.83) | 0.78 | 1.39(0.81-2.37) | 0.23 | 1.14(0.61-2.11) | 0.68 |
| **AGE (year)** |  |  |  |  |  |  |
| ＜60 | reference |  | reference |  | reference |  |
| ≥60 | 3.58(2.37-5.40) | <0.0001 | 3.19(2.20-4.64) | <0.0001 | 3.84(2.12-6.95) | <0.0001 |
| **SUBTYPE** |  |  |  |  |  |  |
| HoR+/HER2- | reference |  | reference |  | reference |  |
| HoR+/HER2+ | 0.57(0.18-1.80) | 0.34 | 0.73(0.41-1.28) | 0.27 | 0.55(0.21-1.45) | 0.22 |
| HoR-/HER2+ | 2.13(0.29-15.64) | 0.46 | 1.31(0.73-2.37) | 0.37 | 1.52(0.76-3.00) | 0.23 |
| HoR-/HER2- | 1.29(0.41-4.06) | 0.66 | 1.32(0.79-2.23) | 0.29 | 1.07(0.59-1.94) | 0.82 |

Abbreviations: HR: hazard ratio; HoR: hormone receptor; HER‐2: human epidermal growth factor receptor‐2
