# Supplementary material for: Impact of adjuvant chemotherapy on T1N0M0 breast cancer patients: a propensity score matching study based on SEER database and external cohort
Source: BMC Cancer. 2022 Aug 8;22:863. doi: 10.1186/s12885-022-09952-z (PMC9358893; doi:10.1186/s12885-022-09952-z)
Supplement: Supplementary file 9 — Additional file 9: Table S6. Multivariable Cox regression analyses of overall survival for molecularsubtypes in T1b breast cancer patients. [file 12885_2022_9952_MOESM9_ESM.docx]

Table S6: Multivariable Cox regression analyses of overall survival for molecular subtypes in T1b breast cancer patients.

| **Variable** | T1b: HoR+/HER2- | | T1b: HoR+/HER2+ | | T1b：HoR-/HER2+ | | T1b：HoR-/HER2- | |
| --- | --- | --- | --- | --- | --- | --- | --- | --- |
|  | **Multivariate Analysis** | | **Multivariate Analysis** | | **Multivariate Analysis** | | **Multivariate Analysis** | |
|  | HR (95%CI) | P-value | HR (95%CI) | P-value | HR (95%CI) | P-value | HR (95%CI) | P-value |
| **GRADE** |  |  |  |  |  |  |  |  |
| I | reference |  | reference |  | reference |  | reference |  |
| II | 1.00(0.88-1.14) | 0.99 | 0.81(0.46-1.44) | 0.48 | 1.72(0.22-13.42) | 0.60 | 2.00(0.62-6.51) | 0.25 |
| III | 1.12(0.89-1.40) | 0.32 | 1.01(0.55-1.86) | 0.98 | 1.68(0.22-12.64) | 0.61 | 2.59(0.82-8.22) | 0.11 |
| **SURGERY** |  |  |  |  |  |  |  |  |
| Breast-conserving | reference |  | reference |  | reference |  | reference |  |
| Total mastectomy | 0.70(0.58-0.84) | <0.0001 | 0.76(0.39-1.49) | 0.43 | 0.41(0.14-1.24) | 0.11 | 1.93(1.01-3.70) | 0.05 |
| Modified radical mastectomy | 0.90(0.70-1.15) | 0.38 | 1.15(0.50-2.64) | 0.74 | 0.79(0.22-2.83) | 0.72 | 1.95(0.92-4.13) | 0.08 |
| **RADIATION** |  |  |  |  |  |  |  |  |
| No | reference |  | reference |  | reference |  | reference |  |
| Yes | 0.40(0.34-0.47) | <0.0001 | 0.60(0.32-1.12) | 0.11 | 0.50(0.17-1.47) | 0.21 | 1.24(0.67-2.31) | 0.50 |
| **CHEMOTHERAPY** |  |  |  |  |  |  |  |  |
| No | reference |  | reference |  | reference |  | reference |  |
| Yes | 1.06(0.81-1.39) | 0.67 | 0.41(0.25-0.69) | <0.0001 | 0.50(0.25-1.02) | 0.01 | 0.53(0.36-0.78) | <0.0001 |
| **AGE (year)** |  |  |  |  |  |  |  |  |
| ＜60 | reference |  | reference |  | reference |  | reference |  |
| ≥60 | 3.95(3.29-4.74) | <0.0001 | 4.96(2.71-9.10) | <0.0001 | 2.04(0.96-4.36) | 0.06 | 2.40(1.53-3.78) | <0.0001 |

Abbreviations: HR: hazard ratio; HoR: hormone receptor; HER‐2: human epidermal growth factor receptor‐2
