# Supplementary material for: Impact of adjuvant chemotherapy on T1N0M0 breast cancer patients: a propensity score matching study based on SEER database and external cohort
Source: BMC Cancer. 2022 Aug 8;22:863. doi: 10.1186/s12885-022-09952-z (PMC9358893; doi:10.1186/s12885-022-09952-z)
Supplement: Supplementary file 10 — Additional file 10: Table S7.Multivariable Cox regression analyses of overall survival for tumorgrades in T1b breast cancer patients. [file 12885_2022_9952_MOESM10_ESM.docx]

Table S7: Multivariable Cox regression analyses of overall survival for tumor grades in T1b breast cancer patients.

| **Variable** | T1b：GRADEⅠ | | T1b：GRADEⅡ | | T1b：GRADE Ⅲ | |
| --- | --- | --- | --- | --- | --- | --- |
|  | **Multivariate Analysis** | | **Multivariate Analysis** | | **Multivariate Analysis** | |
|  | HR (95%CI) | P-value | HR (95%CI) | P-value | HR (95%CI) | P-value |
| **SURGERY** |  |  |  |  |  |  |
| Breast-conserving | reference |  | reference |  | reference |  |
| Total mastectomy | 0.76(0.58-0.99) | 0.04 | 0.66(0.51-0.85) | <0.01 | 1.09(0.70-1.70) | 0.71 |
| Modified radical mastectomy | 0.96(0.67-1.37) | 0.81 | 0.89(0.63-1.24) | 0.47 | 1.33(0.78-2.25) | 0.29 |
| **RADIATION** |  |  |  |  |  |  |
| No | reference |  | reference |  | reference |  |
| Yes | 0.42(0.33-0.53) | <0.0001 | 0.39(0.31-0.49) | <0.0001 | 0.80(0.52-1.21) | 0.29 |
| **CHEMOTHERAPY** |  |  |  |  |  |  |
| No | reference |  | reference |  | reference |  |
| Yes | 1.21(0.77-1.92) | 0.41 | 0.77(0.56-1.06) | 0.11 | 0.52(0.38-0.70) | <0.0001 |
| **AGE (year)** |  |  |  |  |  |  |
| ＜60 | reference |  | reference |  | reference |  |
| ≥60 | 4.80(3.61-6.38) | <0.0001 | 3.80(2.96-4.88) | <0.0001 | 2.42(1.77-3.30) | <0.0001 |
| **SUBTYPE** |  |  |  |  |  |  |
| HoR+/HER2- | reference |  | reference |  | reference |  |
| HoR+/HER2+ | 1.53(0.93-2.53) | 0.09 | 1.24(0.88-1.76) | 0.22 | 1.34(0.88-2.05) | 0.18 |
| HoR-/HER2+ | 0.93(0.13-6.70) | 0.94 | 1.90(1.02-3.52) | 0.04 | 1.78(1.10-2.87) | 0.02 |
| HoR-/HER2- | 0.76(0.24-2.39) | 0.64 | 1.58(1.11-2.25) | 0.01 | 1.86(1.38-2.51) | <0.0001 |

Abbreviations: HR: hazard ratio; HoR: hormone receptor; HER‐2: human epidermal growth factor receptor‐2
