# Supplementary material for: Impact of adjuvant chemotherapy on T1N0M0 breast cancer patients: a propensity score matching study based on SEER database and external cohort
Source: BMC Cancer. 2022 Aug 8;22:863. doi: 10.1186/s12885-022-09952-z (PMC9358893; doi:10.1186/s12885-022-09952-z)
Supplement: Supplementary file 11 — Additional file 11: Table S8. Multivariable Coxregression analyses of overall survival for molecular subtypes in T1c breast cancer patients. [file 12885_2022_9952_MOESM11_ESM.docx]

Table S8: Multivariable Cox regression analyses of overall survival for molecular subtypes in T1c breast cancer patients.

| **Variable** | T1c：HoR+/HER2- | | T1c：HoR+/HER2+ | | T1c：HoR-/HER2+ | | T1c：HoR-/HER2- | |
| --- | --- | --- | --- | --- | --- | --- | --- | --- |
|  | **Multivariate Analysis** | | **Multivariate Analysis** | | **Multivariate Analysis** | | **Multivariate Analysis** | |
|  | HR (95%CI) | P-value | HR (95%CI) | P-value | HR (95%CI) | P-value | HR (95%CI) | P-value |
| **GRADE** |  |  |  |  |  |  |  |  |
| I | reference |  | reference |  | reference |  | reference |  |
| II | 1.06(0.96-1.17) | 0.26 | 1.18(0.74-1.86) | 0.49 | 0.35(0.08-1.51) | 0.16 | 2.19(1.11-4.35) | 0.02 |
| III | 1.50(1.32-1.71) | <0.0001 | 1.15(0.72-1.84) | 0.56 | 0.35(0.08-1.45) | 0.15 | 2.57(1.32-5.00) | 0.01 |
| **SURGERY** |  |  |  |  |  |  |  |  |
| Breast-conserving | reference |  | reference |  | reference |  | reference |  |
| Total mastectomy | 0.50(0.45-0.57) | <0.0001 | 0.66(0.46-0.94) | 0.02 | 0.42(0.23-0.78) | 0.01 | 0.73(0.56-0.97) | 0.03 |
| Modified radical mastectomy | 0.62(0.53-0.73) | <0.0001 | 0.57(0.35-0.94) | 0.03 | 0.47(0.21-1.04) | 0.06 | 0.59(0.40-0.86) | 0.01 |
| **RADIATION** |  |  |  |  |  |  |  |  |
| No | reference |  | reference |  | reference |  | reference |  |
| Yes | 0.29(0.26-0.33) | <0.0001 | 0.38(0.26-0.54) | <0.0001 | 0.44(0.23-0.81) | 0.01 | 0.51(0.39-0.67) | <0.0001 |
| **CHEMOTHERAPY** |  |  |  |  |  |  |  |  |
| No | reference |  | reference |  | reference |  | reference |  |
| Yes | 0.73(0.63-0.86) | <0.0001 | 0.41(0.31-0.54) | <0.0001 | 0.31(0.19-0.50) | <0.0001 | 0.35(0.29-0.43) | <0.0001 |
| **AGE (year)** |  |  |  |  |  |  |  |  |
| ＜60 | reference |  | reference |  | reference |  | reference |  |
| ≥60 | 4.13(3.64-4.69) | <0.0001 | 2.97(2.15-4.09) | <0.0001 | 2.39(1.44-3.98) | <0.0001 | 1.75(1.41-2.18) | <0.0001 |

Abbreviations: HR: hazard ratio; HoR: hormone receptor; HER‐2: human epidermal growth factor receptor‐2
