# Supplementary material for: Impact of adjuvant chemotherapy on T1N0M0 breast cancer patients: a propensity score matching study based on SEER database and external cohort
Source: BMC Cancer. 2022 Aug 8;22:863. doi: 10.1186/s12885-022-09952-z (PMC9358893; doi:10.1186/s12885-022-09952-z)
Supplement: Supplementary file 12 — Additional file 12: Table S9. Multivariable Coxregression analyses of overall survival for tumor grades in T1c breast cancerpatients. [file 12885_2022_9952_MOESM12_ESM.docx]

Table S9: Multivariable Cox regression analyses of overall survival for tumor grades in T1c breast cancer patients.

| **Variable** | T1c：GRADEⅠ | | T1c：GRADEⅡ | | T1c：GRADEⅢ | |
| --- | --- | --- | --- | --- | --- | --- |
|  | **Multivariate Analysis** | | **Multivariate Analysis** | | **Multivariate Analysis** | |
|  | HR (95%CI) | P-value | HR (95%CI) | P-value | HR (95%CI) | P-value |
| **SURGERY** |  |  |  |  |  |  |
| Breast-conserving | reference |  | reference |  | reference |  |
| Total mastectomy | 0.51(0.41-0.62) | <0.0001 | 0.53(0.46-0.62) | <0.0001 | 0.60(0.49-0.72) | <0.0001 |
| Modified radical mastectomy | 0.58(0.44-0.77) | <0.01 | 0.66(0.54-0.81) | <0.0001 | 0.56(0.43-0.73) | <0.0001 |
| **RADIATION** |  |  |  |  |  |  |
| No | reference |  | reference |  | reference |  |
| Yes | 0.27(0.22-0.32) | <0.0001 | 0.31(0.27-0.36) | <0.0001 | 0.41(0.34-0.49) | <0.0001 |
| **CHEMOTHERAPY** |  |  |  |  |  |  |
| No | reference |  | reference |  | reference |  |
| Yes | 0.72(0.48-1.07) | 0.10 | 0.61(0.50-0.73) | <0.0001 | 0.43(0.37-0.50) | <0.0001 |
| **AGE (year)** |  |  |  |  |  |  |
| ＜60 | reference |  | reference |  | reference |  |
| ≥60 | 4.45(3.51-5.65) | <0.0001 | 4.46(3.77-5.28) | <0.0001 | 2.13(1.83-2.48) | <0.0001 |
| **SUBTYPE** |  |  |  |  |  |  |
| HoR+/HER2- | reference |  | reference |  | reference |  |
| HoR+/HER2+ | 1.41(0.91-2.19) | 0.12 | 1.54(1.25-1.90) | <0.0001 | 1.09(0.87-1.37) | 0.47 |
| HoR-/HER2+ | 4.13(0.99-17.14) | 0.05 | 1.65(1.05-2.59) | 0.03 | 1.24(0.93-1.64) | 0.14 |
| HoR-/HER2- | 1.00(0.52-1.95) | 0.99 | 2.20(1.77-2.73) | <0.0001 | 1.93(1.66-2.25) | <0.0001 |

Abbreviations: HR: hazard ratio; HoR: hormone receptor; HER‐2: human epidermal growth factor receptor‐2
