# Supplementary material for: Impact of adjuvant chemotherapy on T1N0M0 breast cancer patients: a propensity score matching study based on SEER database and external cohort
Source: BMC Cancer. 2022 Aug 8;22:863. doi: 10.1186/s12885-022-09952-z (PMC9358893; doi:10.1186/s12885-022-09952-z)
Supplement: Supplementary file 13 — Additional file 13: Table S10. Multivariable Coxregression analyses of overall survival for tumor grades in HoR+/HER2- T1abreast cancer patients. [file 12885_2022_9952_MOESM13_ESM.docx]

Table S10: Multivariable Cox regression analyses of overall survival for tumor grades in HoR+/HER2- T1a breast cancer patients.

| **Variable** | T1a：GRADEⅠ | | T1a：GRADEⅡ | | T1a：GRADE Ⅲ | |
| --- | --- | --- | --- | --- | --- | --- |
|  | **Multivariate Analysis** | | **Multivariate Analysis** | | **Multivariate Analysis** | |
|  | HR (95%CI) | P-value | HR (95%CI) | P-value | HR (95%CI) | P-value |
| **SURGERY** |  |  |  |  |  |  |
| Breast-conserving | reference |  | reference |  | reference |  |
| Total mastectomy | 0.79(0.52-1.22) | 0.29 | 0.53(0.33-0.84) | 0.01 | 0.37(0.12-1.12) | 0.08 |
| Modified radical mastectomy | 1.11(0.61-2.00) | 0.73 | 0.56(0.28-1.11) | 0.10 | 0.32(0.07-1.36) | 0.12 |
| **RADIATION** |  |  |  |  |  |  |
| No | reference |  | reference |  | reference |  |
| Yes | 0.37(0.25-0.56) | <0.0001 | 0.33(0.21-0.50) | <0.0001 | 0.16(0.05-0.48) | <0.01 |
| **CHEMOTHERAPY** |  |  |  |  |  |  |
| No | reference |  | reference |  | reference |  |
| Yes | 1.15(0.42-3.11) | 0.79 | 3.23(1.81-5.78) | <0.0001 | 3.26(1.25-8.48) | 0.02 |
| **AGE (year)** |  |  |  |  |  |  |
| ＜60 | reference |  | reference |  | reference |  |
| ≥60 | 3.88(2.52-5.96) | <0.0001 | 4.03(2.53-6.41) | <0.0001 | 3.47(1.35-8.92) | 0.01 |

Abbreviations: HoR: hormone receptor; HER‐2: human epidermal growth factor receptor‐2; HR: hazard ratio
