# Supplementary material for: Impact of adjuvant chemotherapy on T1N0M0 breast cancer patients: a propensity score matching study based on SEER database and external cohort
Source: BMC Cancer. 2022 Aug 8;22:863. doi: 10.1186/s12885-022-09952-z (PMC9358893; doi:10.1186/s12885-022-09952-z)
Supplement: Supplementary file 14 — Additional file 14: Table S11. Multivariable Cox regression analyses of overall survival for tumorgrades in HoR+/HER2+ T1abreast cancer patients. [file 12885_2022_9952_MOESM14_ESM.docx]

Table S11: Multivariable Cox regression analyses of overall survival for tumor grades in HoR+/HER2+ T1a breast cancer patients.

| **Variable** | T1a：GRADEⅠ | | T1a：GRADEⅡ | | T1a：GRADE Ⅲ | |
| --- | --- | --- | --- | --- | --- | --- |
|  | **Multivariate Analysis** | | **Multivariate Analysis** | | **Multivariate Analysis** | |
|  | HR (95%CI) | P-value | HR (95%CI) | P-value | HR (95%CI) | P-value |
| **SURGERY** |  |  |  |  |  |  |
| Breast-conserving | reference |  | reference |  | reference |  |
| Total mastectomy | 2.27(0.02-217.64) | 0.72 | 0.87(0.14-5.22) | 0.88 | 3.44(0.09-134.65) | 0.51 |
| Modified radical mastectomy | - | - | 1.32(0.15-11.57) | 0.80 | - | - |
| **RADIATION** |  |  |  |  |  |  |
| No | reference |  | reference |  | reference |  |
| Yes | 2.71(0.03-257.29) | 0.67 | 0.52(0.09-3.05) | 0.46 | 0.57(0.01-22.25) | 0.76 |
| **CHEMOTHERAPY** |  |  |  |  |  |  |
| No | reference |  | reference |  | reference |  |
| Yes | - | - | 0.67(0.14-3.08) | 0.60 | 0.83(0.09-7.42) | 0.86 |
| **AGE (year)** |  |  |  |  |  |  |
| ＜60 | reference |  | reference |  | reference |  |
| ≥60 | 0.41(0.04-4.60) | 0.47 | 6.27(1.67-23.45) | 0.01 | - | - |

Abbreviations: HoR: hormone receptor; HER‐2: human epidermal growth factor receptor‐2; HR: hazard ratio
