# Supplementary material for: Impact of adjuvant chemotherapy on T1N0M0 breast cancer patients: a propensity score matching study based on SEER database and external cohort
Source: BMC Cancer. 2022 Aug 8;22:863. doi: 10.1186/s12885-022-09952-z (PMC9358893; doi:10.1186/s12885-022-09952-z)
Supplement: Supplementary file 15 — Additional file 15: Table S12. Multivariable Cox regression analyses of overall survival for tumorgrades in HoR-/HER2+ T1a breast cancer patients. [file 12885_2022_9952_MOESM15_ESM.docx]

Table S12: Multivariable Cox regression analyses of overall survival for tumor grades in HoR-/HER2+ T1a breast cancer patients.

| **Variable** | T1A：GRADEⅠ | | T1a：GRADEⅡ | | T1a：GRADE Ⅲ | |
| --- | --- | --- | --- | --- | --- | --- |
|  | **Multivariate Analysis** | | **Multivariate Analysis** | | **Multivariate Analysis** | |
|  | HR (95%CI) | P-value | HR (95%CI) | P-value | HR (95%CI) | P-value |
| **SURGERY** |  |  |  |  |  |  |
| Breast-conserving | reference |  | reference |  | reference |  |
| Total mastectomy | - | - | 0.46(0.06-3.72) | 0.46 | 6.26(1.00-39.28) | 0.05 |
| Modified radical mastectomy | - | - | 0.58(0.05-6.26) | 0.65 | - | - |
| **RADIATION** |  |  |  |  |  |  |
| No | reference |  | reference |  | reference |  |
| Yes | 21.13(21.13-21.13) | <0.0001 | 0.57(0.07-4.59) | 0.59 | 2.89(0.62-13.51) | 0.18 |
| **CHEMOTHERAPY** |  |  |  |  |  |  |
| No | reference |  | reference |  | reference |  |
| Yes | - | - | 0.19(0.02-1.51) | 0.12 | 0.64(0.16-2.47) | 0.51 |
| **AGE (year)** |  |  |  |  |  |  |
| ＜60 | reference |  | reference |  | reference |  |
| ≥60 | 2.55(2.55-2.55) | <0.0001 | 1.13(0.36-3.58) | 0.83 | 5.32(1.43-19.82) | 0.01 |

Abbreviations: HoR: hormone receptor; HER‐2: human epidermal growth factor receptor‐2; HR: hazard ratio
