# Supplementary material for: Impact of adjuvant chemotherapy on T1N0M0 breast cancer patients: a propensity score matching study based on SEER database and external cohort
Source: BMC Cancer. 2022 Aug 8;22:863. doi: 10.1186/s12885-022-09952-z (PMC9358893; doi:10.1186/s12885-022-09952-z)
Supplement: Supplementary file 16 — Additional file 16: Table S13. Multivariable Cox regression analyses of overall survival for tumorgrades in HoR-/HER2- T1a breast cancer patients. [file 12885_2022_9952_MOESM16_ESM.docx]

Table S13: Multivariable Cox regression analyses of overall survival for tumor grades in HoR-/HER2- T1a breast cancer patients.

| **Variable** | T1a：GRADEⅠ | | T1a：GRADEⅡ | | T1a：GRADE Ⅲ | |
| --- | --- | --- | --- | --- | --- | --- |
|  | **Multivariate Analysis** | | **Multivariate Analysis** | | **Multivariate Analysis** | |
|  | HR (95%CI) | P-value | HR (95%CI) | P-value | HR (95%CI) | P-value |
| **SURGERY** |  |  |  |  |  |  |
| Breast-conserving | reference |  | reference |  | reference |  |
| Total mastectomy | - | - | 0.53(0.14-1.97) | 0.35 | 0.48(0.14-1.60) | 0.23 |
| Modified radical mastectomy | - | - | 0.27(0.03-2.65) | 0.26 | 0.18(0.02-1.60) | 0.12 |
| **RADIATION** |  |  |  |  |  |  |
| No | reference |  | reference |  | reference |  |
| Yes | 0.06(0.01-0.70) | 0.02 | 0.18(0.04-0.78) | 0.02 | 0.24(0.07-0.80) | 0.02 |
| **CHEMOTHERAPY** |  |  |  |  |  |  |
| No | reference |  | reference |  | reference |  |
| Yes | - | - | 0.33(0.04-2.63) | 0.30 | 0.63(0.21-1.91) | 0.42 |
| **AGE (year)** |  |  |  |  |  |  |
| ＜60 | reference |  | reference |  | reference |  |
| ≥60 | - | - | 0.81(0.28-2.29) | 0.68 | 1.93(0.73-5.13) | 0.19 |

Abbreviations: HoR: hormone receptor; HER‐2: human epidermal growth factor receptor‐2; HR: hazard ratio
