# Supplementary material for: Impact of adjuvant chemotherapy on T1N0M0 breast cancer patients: a propensity score matching study based on SEER database and external cohort
Source: BMC Cancer. 2022 Aug 8;22:863. doi: 10.1186/s12885-022-09952-z (PMC9358893; doi:10.1186/s12885-022-09952-z)
Supplement: Supplementary file 17 — Additional file 17: Table S14. Multivariable Coxregression analyses of overall survival for tumor grades in HoR+/HER2- T1b breastcancer patients. [file 12885_2022_9952_MOESM17_ESM.docx]

Table S14: Multivariable Cox regression analyses of overall survival for tumor grades in HoR+/HER2- T1b breast cancer patients.

| **Variable** | T1b：GRADEⅠ | | T1b：GRADEⅡ | | T1b：GRADE Ⅲ | |
| --- | --- | --- | --- | --- | --- | --- |
|  | **Multivariate Analysis** | | **Multivariate Analysis** | | **Multivariate Analysis** | |
|  | HR (95%CI) | P-value | HR (95%CI) | P-value | HR (95%CI) | P-value |
| **SURGERY** |  |  |  |  |  |  |
| Breast-conserving | reference |  | reference |  | reference |  |
| Total mastectomy | 0.74(0.56-0.98) | 0.03 | 0.65(0.49-0.85) | <0.01 | 0.78(0.41-1.48) | 0.45 |
| Modified radical mastectomy | 0.96(0.66-1.38) | 0.81 | 0.77(0.53-1.12) | 0.17 | 1.29(0.61-2.74) | 0.51 |
| **RADIATION** |  |  |  |  |  |  |
| No | reference |  | reference |  | reference |  |
| Yes | 0.42(0.33-0.53) | <0.0001 | 0.37(0.29-0.47) | <0.0001 | 0.49(0.27-0.89) | 0.02 |
| **CHEMOTHERAPY** |  |  |  |  |  |  |
| No | reference |  | reference |  | reference |  |
| Yes | 1.62(1.02-2.57) | 0.04 | 0.98(0.64-1.49) | 0.91 | 0.71(0.42-1.22) | 0.22 |
| **AGE (year)** |  |  |  |  |  |  |
| ＜60 | reference |  | reference |  | reference |  |
| ≥60 | 4.59(3.45-6.12) | <0.0001 | 4.15(3.13-5.51) | <0.0001 | 2.01(1.26-3.21) | <0.01 |

Abbreviations: HoR: hormone receptor; HER‐2: human epidermal growth factor receptor‐2; HR: hazard ratio
