# Supplementary material for: Impact of adjuvant chemotherapy on T1N0M0 breast cancer patients: a propensity score matching study based on SEER database and external cohort
Source: BMC Cancer. 2022 Aug 8;22:863. doi: 10.1186/s12885-022-09952-z (PMC9358893; doi:10.1186/s12885-022-09952-z)
Supplement: Supplementary file 18 — Additional file 18: Table S15. Multivariable Coxregression analyses of overall survival for tumor grades in HoR+/HER2+ T1b breast cancer patients. [file 12885_2022_9952_MOESM18_ESM.docx]

Table S15: Multivariable Cox regression analyses of overall survival for tumor grades in HoR+/HER2+ T1b breast cancer patients.

| **Variable** | T1b：GRADEⅠ | | T1b：GRADEⅡ | | T1b：GRADE Ⅲ | |
| --- | --- | --- | --- | --- | --- | --- |
|  | **Multivariate Analysis** | | **Multivariate Analysis** | | **Multivariate Analysis** | |
|  | HR (95%CI) | P-value | HR (95%CI) | P-value | HR (95%CI) | P-value |
| **SURGERY** |  |  |  |  |  |  |
| Breast-conserving | reference |  | reference |  | reference |  |
| Total mastectomy | 0.79(0.23-2.73) | 0.71 | 0.55(0.21-1.43) | 0.22 | 1.53(0.37-6.36) | 0.56 |
| Modified radical mastectomy | 0.63(0.11-3.64) | 0.60 | 1.66(0.58-4.72) | 0.34 | 0.68(0.06-7.19) | 0.75 |
| **RADIATION** |  |  |  |  |  |  |
| No | reference |  | reference |  | reference |  |
| Yes | 0.19(0.05-0.77) | 0.02 | 0.42(0.17-1.03) | 0.06 | 2.17(0.54-8.68) | 0.27 |
| **CHEMOTHERAPY** |  |  |  |  |  |  |
| No | reference |  | reference |  | reference |  |
| Yes | 0.18(0.02-1.39) | 0.10 | 0.50(0.24-1.03) | 0.06 | 0.36(0.16-0.80) | 0.01 |
| **AGE (year)** |  |  |  |  |  |  |
| ＜60 | reference |  | reference |  | reference |  |
| ≥60 | 14.86(1.96-112.55) | 0.01 | 4.58(1.86-11.29) | <0.01 | 3.67(1.45-9.32) | 0.01 |

Abbreviations: HoR: hormone receptor; HER‐2: human epidermal growth factor receptor‐2; HR: hazard ratio
