# Supplementary material for: Impact of adjuvant chemotherapy on T1N0M0 breast cancer patients: a propensity score matching study based on SEER database and external cohort
Source: BMC Cancer. 2022 Aug 8;22:863. doi: 10.1186/s12885-022-09952-z (PMC9358893; doi:10.1186/s12885-022-09952-z)
Supplement: Supplementary file 19 — Additional file 19: Table S16. Multivariable Coxregression analyses of overall survival for tumor grades in HoR-/HER2+ T1b breast cancer patients. [file 12885_2022_9952_MOESM19_ESM.docx]

Table S16: Multivariable Cox regression analyses of overall survival for tumor grades in HoR-/HER2+ T1b breast cancer patients.

| **Variable** | T1b：GRADEⅠ | | T1b：GRADEⅡ | | T1b：GRADE Ⅲ | |
| --- | --- | --- | --- | --- | --- | --- |
|  | **Multivariate Analysis** | | **Multivariate Analysis** | | **Multivariate Analysis** | |
|  | HR (95%CI) | P-value | HR (95%CI) | P-value | HR (95%CI) | P-value |
| **SURGERY** |  |  |  |  |  |  |
| Breast-conserving | reference |  | reference |  | reference |  |
| Total mastectomy | - | - | 0.29(0.03-2.59) | 0.27 | 0.46(0.13-1.69) | 0.24 |
| Modified radical mastectomy | - | - | 0.48(0.03-7.52) | 0.60 | 0.92(0.21-4.01) | 0.91 |
| **RADIATION** |  |  |  |  |  |  |
| No | reference |  | reference |  | reference |  |
| Yes | - | - | 0.51(0.06-4.15) | 0.53 | 0.46(0.12-1.72) | 0.25 |
| **CHEMOTHERAPY** |  |  |  |  |  |  |
| No | reference |  | reference |  | reference |  |
| Yes | - | - | 0.93(0.25-3.44) | 0.91 | 0.34(0.14-0.84) | 0.02 |
| **AGE (year)** |  |  |  |  |  |  |
| ＜60 | reference |  | reference |  | reference |  |
| ≥60 | - | - | 1.89(0.50-7.06) | 0.34 | 2.18(0.84-5.67) | 0.11 |

Abbreviations: HoR: hormone receptor; HER‐2: human epidermal growth factor receptor‐2; HR: hazard ratio
