# Supplementary material for: Impact of adjuvant chemotherapy on T1N0M0 breast cancer patients: a propensity score matching study based on SEER database and external cohort
Source: BMC Cancer. 2022 Aug 8;22:863. doi: 10.1186/s12885-022-09952-z (PMC9358893; doi:10.1186/s12885-022-09952-z)
Supplement: Supplementary file 20 — Additional file 20: Table S17. MultivariableCox regression analysesof overall survival for tumor grades in HoR-/HER2- T1b breast cancer patients. [file 12885_2022_9952_MOESM20_ESM.docx]

Table S17: Multivariable Cox regression analyses of overall survival for tumor grades in HoR-/HER2- T1b breast cancer patients.

| **Variable** | T1b：GRADEⅠ | | T1b：GRADEⅡ | | T1b：GRADE Ⅲ | |
| --- | --- | --- | --- | --- | --- | --- |
|  | **Multivariate Analysis** | | **Multivariate Analysis** | | **Multivariate Analysis** | |
|  | HR (95%CI) | P-value | HR (95%CI) | P-value | HR (95%CI) | P-value |
| **SURGERY** |  |  |  |  |  |  |
| Breast-conserving | reference |  | reference |  | reference |  |
| Total mastectomy | 1.49(0.03-75.54) | 0.84 | 1.95(0.58-6.49) | 0.28 | 1.94(0.88-4.24) | 0.10 |
| Modified radical mastectomy | - | - | 2.92(0.77-11.06) | 0.11 | 1.70(0.67-4.29) | 0.26 |
| **RADIATION** |  |  |  |  |  |  |
| No | reference |  | reference |  | reference |  |
| Yes | 1.77(0.03-89.80) | 0.78 | 1.08(0.34-3.41) | 0.90 | 1.28(0.60-2.72) | 0.53 |
| **CHEMOTHERAPY** |  |  |  |  |  |  |
| No | reference |  | reference |  | reference |  |
| Yes | - | - | 0.54(0.25-1.14) | 0.11 | 0.54(0.34-0.85) | 0.01 |
| **AGE (year)** |  |  |  |  |  |  |
| ＜60 | reference |  | reference |  | reference |  |
| ≥60 | - | 1.00 | 1.58(0.67-3.74) | 0.30 | 2.67(1.56-4.55) | <0.01 |

Abbreviations: HoR: hormone receptor; HER‐2: human epidermal growth factor receptor‐2; HR: hazard ratio
