# Supplementary material for: Impact of adjuvant chemotherapy on T1N0M0 breast cancer patients: a propensity score matching study based on SEER database and external cohort
Source: BMC Cancer. 2022 Aug 8;22:863. doi: 10.1186/s12885-022-09952-z (PMC9358893; doi:10.1186/s12885-022-09952-z)
Supplement: Supplementary file 21 — Additional file 21: Table S18. Multivariable Cox regression analyses of overall survival for tumorgrades in HoR+/HER2- T1c breast cancer patients. [file 12885_2022_9952_MOESM21_ESM.docx]

Table S18: Multivariable Cox regression analyses of overall survival for tumor grades in HoR+/HER2- T1c breast cancer patients .

| **Variable** | T1c：GRADEⅠ | | T1c：GRADEⅡ | | T1c：GRADEⅢ | |
| --- | --- | --- | --- | --- | --- | --- |
|  | **Multivariate Analysis** | | **Multivariate Analysis** | | **Multivariate Analysis** | |
|  | HR (95%CI) | P-value | HR (95%CI) | P-value | HR (95%CI) | P-value |
| **SURGERY** |  |  |  |  |  |  |
| Breast-conserving | reference |  | reference |  | reference |  |
| Total mastectomy | 0.50(0.40-0.61) | <0.0001 | 0.52(0.44-0.62) | <0.0001 | 0.49(0.36-0.65) | <0.0001 |
| Modified radical mastectomy | 0.59(0.45-0.79) | <0.01 | 0.66(0.53-0.82) | <0.01 | 0.59(0.40-0.85) | 0.01 |
| **RADIATION** |  |  |  |  |  |  |
| No | reference |  | reference |  | reference |  |
| Yes | 0.26(0.21-0.32) | <0.0001 | 0.30(0.25-0.35) | <0.0001 | 0.36(0.28-0.47) | <0.0001 |
| **CHEMOTHERAPY** |  |  |  |  |  |  |
| No | reference |  | reference |  | reference |  |
| Yes | 0.75(0.48-1.17) | 0.20 | 0.78(0.61-0.99) | 0.04 | 0.63(0.50-0.79) | <0.0001 |
| **AGE (year)** |  |  |  |  |  |  |
| ＜60 | reference |  | reference |  | reference |  |
| ≥60 | 4.54(3.55-5.80) | <0.0001 | 5.19(4.27-6.31) | <0.0001 | 2.41(1.90-3.05) | <0.0001 |

Abbreviations: HoR: hormone receptor; HER2: human epidermal growth factor receptor‐2; HR: hazard ratio
