# Supplementary material for: Impact of adjuvant chemotherapy on T1N0M0 breast cancer patients: a propensity score matching study based on SEER database and external cohort
Source: BMC Cancer. 2022 Aug 8;22:863. doi: 10.1186/s12885-022-09952-z (PMC9358893; doi:10.1186/s12885-022-09952-z)
Supplement: Supplementary file 22 — Additional file 22: Table S19. Multivariable Cox regression analyses of overall survival fortumor grades in HoR+/HER2+ T1c breast cancer patients. [file 12885_2022_9952_MOESM22_ESM.docx]

Table S19: Multivariable Cox regression analyses of overall survival for tumor grades in HoR+/HER2+ T1c breast cancer patients.

| **Variable** | T1c：GRADEⅠ | | T1c：GRADEⅡ | | T1c：GRADEⅢ | |
| --- | --- | --- | --- | --- | --- | --- |
|  | **Multivariate Analysis** | | **Multivariate Analysis** | | **Multivariate Analysis** | |
|  | HR (95%CI) | P-value | HR (95%CI) | P-value | HR (95%CI) | P-value |
| **SURGERY** |  |  |  |  |  |  |
| Breast-conserving | reference |  | reference |  | reference |  |
| Total mastectomy | 0.60(0.19-1.87) | 0.38 | 0.56(0.33-0.94) | 0.03 | 0.79(0.46-1.39) | 0.42 |
| Modified radical mastectomy | 0.25(0.03-2.17) | 0.21 | 0.66(0.35-1.25) | 0.20 | 0.52(0.22-1.24) | 0.14 |
| **RADIATION** |  |  |  |  |  |  |
| No | reference |  | reference |  | reference |  |
| Yes | 0.38(0.12-1.19) | 0.10 | 0.32(0.19-0.54) | <0.0001 | 0.47(0.27-0.83) | 0.01 |
| **CHEMOTHERAPY** |  |  |  |  |  |  |
| No | reference |  | reference |  | reference |  |
| Yes | 0.59(0.22-1.56) | 0.28 | 0.47(0.32-0.70) | <0.0001 | 0.32(0.21-0.49) | <0.0001 |
| **AGE (year)** |  |  |  |  |  |  |
| ＜60 | reference |  | reference |  | reference |  |
| ≥60 | 2.89(0.96-8.73) | 0.06 | 2.87(1.80-4.59) | <0.0001 | 3.07(1.89-4.96) | <0.0001 |

Abbreviations: HoR: hormone receptor; HER‐2: human epidermal growth factor receptor‐2; HR: hazard ratio
