# Supplementary material for: Impact of adjuvant chemotherapy on T1N0M0 breast cancer patients: a propensity score matching study based on SEER database and external cohort
Source: BMC Cancer. 2022 Aug 8;22:863. doi: 10.1186/s12885-022-09952-z (PMC9358893; doi:10.1186/s12885-022-09952-z)
Supplement: Supplementary file 23 — Additional file 23: Table S20. Multivariable Cox regression analyses of overall survival for tumorgrades in HoR-/HER2+ T1c breast cancer patients. [file 12885_2022_9952_MOESM23_ESM.docx]

Table S20: Multivariable Cox regression analyses of overall survival for tumor grades in HoR-/HER2+ T1c breast cancer patients.

| **Variable** | T1c：GRADEⅠ | | T1c：GRADEⅡ | | T1c：GRADEⅢ | |
| --- | --- | --- | --- | --- | --- | --- |
|  | **Multivariate Analysis** | | **Multivariate Analysis** | | **Multivariate Analysis** | |
|  | HR (95%CI) | P-value | HR (95%CI) | P-value | HR (95%CI) | P-value |
| **SURGERY** |  |  |  |  |  |  |
| Breast-conserving | reference |  | reference |  | reference |  |
| Total mastectomy | 3.54(0.18-69.80) | 0.41 | 0.71(0.19-2.64) | 0.61 | 0.34(0.17-0.69) | <0.01 |
| Modified radical mastectomy | - | - | 0.34(0.04-3.33) | 0.36 | 0.50(0.21-1.18) | 0.12 |
| **RADIATION** |  |  |  |  |  |  |
| No | reference |  | reference |  | reference |  |
| Yes | - | - | 0.97(0.27-3.48) | 0.96 | 0.32(0.15-0.67) | <0.01 |
| **CHEMOTHERAPY** |  |  |  |  |  |  |
| No | reference |  | reference |  | reference |  |
| Yes | 0.28(0.01-5.57) | 0.41 | 0.24(0.09-0.64) | <0.01 | 0.36(0.20-0.64) | <0.0001 |
| **AGE (year)** |  |  |  |  |  |  |
| ＜60 | reference |  | reference |  | reference |  |
| ≥60 | - | - | 3.58(0.97-13.21) | 0.06 | 2.03(1.15-3.59) | 0.01 |

Abbreviations: HoR: hormone receptor; HER‐2: human epidermal growth factor receptor‐2; HR: hazard ratio
