# Supplementary material for: Impact of adjuvant chemotherapy on T1N0M0 breast cancer patients: a propensity score matching study based on SEER database and external cohort
Source: BMC Cancer. 2022 Aug 8;22:863. doi: 10.1186/s12885-022-09952-z (PMC9358893; doi:10.1186/s12885-022-09952-z)
Supplement: Supplementary file 24 — Additional file 24: Table S21. Multivariable Cox regression analysesof overall survival for tumor grades in HoR-/HER2- T1c breast cancer patients. [file 12885_2022_9952_MOESM24_ESM.docx]

Table S21: Multivariable Cox regression analyses of overall survival for tumor grades in HoR-/HER2- T1c breast cancer patients.

| **Variable** | T1c：GRADEⅠ | | T1c：GRADEⅡ | | T1c：GRADEⅢ | |
| --- | --- | --- | --- | --- | --- | --- |
|  | **Multivariate Analysis** | | **Multivariate Analysis** | | **Multivariate Analysis** | |
|  | HR (95%CI) | P-value | HR (95%CI) | P-value | HR (95%CI) | P-value |
| **SURGERY** |  |  |  |  |  |  |
| Breast-conserving | reference |  | reference |  | reference |  |
| Total mastectomy | 1.06(0.18-6.44) | 0.95 | 0.58(0.31-1.08) | 0.09 | 0.78(0.57-1.08) | 0.13 |
| Modified radical mastectomy | 0.70(0.06-7.60) | 0.77 | 0.70(0.32-1.55) | 0.38 | 0.56(0.36-0.87) | 0.01 |
| **RADIATION** |  |  |  |  |  |  |
| No | reference |  | reference |  | reference |  |
| Yes | 0.62(0.11-3.64) | 0.60 | 0.55(0.30-0.98) | 0.04 | 0.50(0.36-0.69) | <0.0001 |
| **CHEMOTHERAPY** |  |  |  |  |  |  |
| No | reference |  | reference |  | reference |  |
| Yes | 0.70(0.14-3.53) | 0.67 | 0.39(0.25-0.60) | <0.0001 | 0.34(0.27-0.43) | <0.0001 |
| **AGE (year)** |  |  |  |  |  |  |
| ＜60 | reference |  | reference |  | reference |  |
| ≥60 | 3.33(0.39-28.46) | 0.27 | 1.92(1.11-3.31) | 0.02 | 1.71(1.34-2.17) | <0.0001 |

Abbreviations: HoR: hormone receptor; HER‐2: human epidermal growth factor receptor‐2; HR: hazard ratio
