# Supplementary material for: Impact of adjuvant chemotherapy on T1N0M0 breast cancer patients: a propensity score matching study based on SEER database and external cohort
Source: BMC Cancer. 2022 Aug 8;22:863. doi: 10.1186/s12885-022-09952-z (PMC9358893; doi:10.1186/s12885-022-09952-z)
Supplement: Supplementary file 25 — Additional file 25: Table S22. Demographic and clinicalcharacteristics of the included T1N0M0 breast cancer patients in Northern Jiangsu People’sHospital. [file 12885_2022_9952_MOESM25_ESM.docx]

Table S22: Demographic and clinical characteristics of the included T1N0M0 breast cancer patients in Northern Jiangsu People’s Hospital.

| Demographic and Clinical Characteristic | T1a | T1b | T1c | P-value |
| --- | --- | --- | --- | --- |
|  | N=98 | N=130 | N=317 |  |
| **GRADE** |  |  |  | p<0.001 |
| I | 38 (38.78%) | 26 (20.00%) | 60 (18.93%) |  |
| II | 52 (53.06%) | 84 (64.62%) | 160 (50.47%) |  |
| III | 8 (8.16%) | 20 (15.38%) | 97 (30.60%) |  |
| **SURGERY** |  |  |  | p<0.001 |
| Breast-conserving | 14 (14.29%) | 16 (12.31%) | 34 (10.73%) |  |
| Total mastectomy | 22 (22.45%) | 22 (16.92%) | 34 (10.73%) |  |
| Modified radical mastectomy | 62 (63.27%) | 92 (70.77%) | 249 (78.55%) |  |
| **RADIATION** |  |  |  | p<0.001 |
| No | 84 (85.71%) | 116 (89.23%) | 273 (86.12%) |  |
| Yes | 14 (14.29%) | 14 (10.77%) | 44 (13.88%) |  |
| **CHEMOTHERAPY** |  |  |  | p<0.001 |
| No | 54 (55.10%) | 42 (32.31%) | 77 (24.29%) |  |
| Yes | 44 (44.90%) | 88 (67.69%) | 240 (75.71%) |  |
| **SUBTYPE** |  |  |  | p<0.001 |
| HoR+/HER2- | 58 (59.18%) | 68 (52.31%) | 144 (45.43%) |  |
| HoR+/HER2+ | 16 (16.33%) | 18 (13.85%) | 62 (19.56%) |  |
| HoR-/HER2+ | 18 (18.37%) | 18 (13.85%) | 48 (15.14%) |  |
| HoR-/HER2- | 6 (6.12%) | 26 (20.00%) | 63 (19.87%) |  |
| **AGE (year)** |  |  |  | p<0.001 |
| ＜60 | 60 (61.22%) | 98 (75.38%) | 258 (81.39%) |  |
| ≥60 | 38 (38.78%) | 32 (24.62%) | 59 (18.61%) |  |

Abbreviations: HoR: hormone receptor; HER‐2: human epidermal growth factor receptor‐2
