# Supplementary material for: Impact of adjuvant chemotherapy on T1N0M0 breast cancer patients: a propensity score matching study based on SEER database and external cohort
Source: BMC Cancer. 2022 Aug 8;22:863. doi: 10.1186/s12885-022-09952-z (PMC9358893; doi:10.1186/s12885-022-09952-z)
Supplement: Supplementary file 26 — Additional file 26: Table S23. Univariableand multivariable Cox regression analyses of overallsurvival for T1a breast cancer patients in Northern Jiangsu People’s Hospital. [file 12885_2022_9952_MOESM26_ESM.docx]

Table S23: Univariable and multivariable Cox regression analyses of overall survival for T1a breast cancer patients in Northern Jiangsu People’s Hospital.

| Variables | T1a | | | |
| --- | --- | --- | --- | --- |
|  | **Univariate Analysis** | | **Multivariate Analysis** | |
|  | HR (95%CI) | P-value | HR (95%CI) | P-value |
| **GRADE** |  |  |  |  |
| I | reference |  | reference |  |
| II | 3.02(0.64-14.26) | 0.16 | 1.86(0.32-10.75) | 0.49 |
| III | 6.44(0.89-46.51) | 0.06 | 5.72(0.64-51.42) | 0.12 |
| **SURGERY** |  |  |  |  |
| Breast-conserving | reference |  | reference |  |
| Total mastectomy | - | - | - | - |
| Modified radical mastectomy | 0.04(0.01-0.21) | <0.0001 | 0.02(0.00-0.26) | <0.01 |
| **RADIATION** |  |  |  |  |
| No | reference |  | reference |  |
| Yes | - | - | - | - |
| **CHEMOTHERAPY** |  |  |  |  |
| No | reference |  | reference |  |
| Yes | 0.51(0.15-1.73) | 0.28 | 3.51(0.11-108.05) | 0.47 |
| **SUBTYPE** |  |  |  |  |
| HoR+/HER2- | reference |  | reference |  |
| HoR+/HER2+ | 0.43(0.07-2.50) | 0.35 | - | - |
| HoR-/HER2+ | - | - | - | - |
| HoR-/HER2- | 0.87(0.12-6.11) | 0.89 | - | - |
| **AGE (year)** |  |  |  |  |
| ＜60 | reference |  | reference |  |
| ≥60 | 6.63(1.41-31.27) | 0.02 | 1.23(0.04-35.66) | 0.90 |

Abbreviations: HR: hazard ratio; HoR: hormone receptor; HER‐2: human epidermal growth factor receptor‐2
