# Supplementary material for: Impact of adjuvant chemotherapy on T1N0M0 breast cancer patients: a propensity score matching study based on SEER database and external cohort
Source: BMC Cancer. 2022 Aug 8;22:863. doi: 10.1186/s12885-022-09952-z (PMC9358893; doi:10.1186/s12885-022-09952-z)
Supplement: Supplementary file 27 — Additional file 27: Table S24. Univariable and multivariable Cox regression analyses ofoverall survival for T1b breast cancer patients in Northern Jiangsu People’s Hospital. [file 12885_2022_9952_MOESM27_ESM.docx]

Table S24: Univariable and multivariable Cox regression analyses of overall survival for T1b breast cancer patients in Northern Jiangsu People’s Hospital.

| Variables | T1b | | | |
| --- | --- | --- | --- | --- |
|  | **Univariate Analysis** | | **Multivariate Analysis** | |
|  | HR (95%CI) | P-value | HR (95%CI) | P-value |
| **GRADE** |  |  |  |  |
| I | reference |  | reference |  |
| II | - | - | - | - |
| III | - | - | - | - |
| **SURGERY** |  |  |  |  |
| Breast-conserving | reference |  | reference |  |
| Total mastectomy | - | - | - | - |
| Modified radical mastectomy | 1.70(0.39-7.33) | 0.48 | - | - |
| **RADIATION** |  |  |  |  |
| No | reference |  | reference |  |
| Yes | 1.05(0.24-4.58) | 0.95 | - | - |
| **CHEMOTHERAPY** |  |  |  |  |
| No | reference |  | reference |  |
| Yes | 0.15(0.06-0.38) | <0.0001 | 0.02(0.00-0.09) | <0.0001 |
| **SUBTYPE** |  |  |  |  |
| HoR+/HER2- | reference |  | reference |  |
| HoR+/HER2+ | 3.72(0.52-26.48) | 0.19 | 36.09(3.34-390.22) | <0.01 |
| HoR-/HER2+ | 15.27(3.18-73.28) | <0.01 | 155.19(17.70-1360.86) | <0.0001 |
| HoR-/HER2- | 28.61(5.84-140.05) | <0.0001 | 703.14(53.11-9308.83) | <0.0001 |
| **AGE (year)** |  |  |  |  |
| ＜60 | reference |  | reference |  |
| ≥60 | 3.02(1.30-7.02) | 0.01 | 2.53(0.71-8.99) | 0.15 |

Abbreviations: HR: hazard ratio; HoR: hormone receptor; HER‐2: human epidermal growth factor receptor‐2
