# Supplementary material for: Impact of adjuvant chemotherapy on T1N0M0 breast cancer patients: a propensity score matching study based on SEER database and external cohort
Source: BMC Cancer. 2022 Aug 8;22:863. doi: 10.1186/s12885-022-09952-z (PMC9358893; doi:10.1186/s12885-022-09952-z)
Supplement: Supplementary file 28 — Additional file 28: Table S25. Univariable and multivariable Cox regression analyses ofoverall survival for T1c breast cancer patients in Northern Jiangsu People’sHospital. [file 12885_2022_9952_MOESM28_ESM.docx]

Table S25: Univariable and multivariable Cox regression analyses of overall survival for T1c breast cancer patients in Northern Jiangsu People’s Hospital.

| Variables | T1c | | | |
| --- | --- | --- | --- | --- |
|  | **Univariate Analysis** | | **Multivariate Analysis** | |
|  | HR (95%CI) | P-value | HR (95%CI) | P-value |
| **GRADE** |  |  |  |  |
| I | reference |  | reference |  |
| II | 2.17(0.83-5.70) | 0.11 | 3.09(1.15-8.31) | 0.03 |
| III | 5.46(2.13-14.02) | <0.01 | 9.04(3.38-24.15) | <0.0001 |
| **SURGERY** |  |  |  |  |
| Breast-conserving | reference |  | reference |  |
| Total mastectomy | 0.40(0.14-1.13) | 0.08 | 0.94(0.31-2.80) | 0.90 |
| Modified radical mastectomy | 0.50(0.27-0.94) | 0.03 | 0.81(0.41-1.61) | 0.54 |
| **RADIATION** |  |  |  |  |
| No | reference |  | reference |  |
| Yes | 0.53(0.21-1.33) | 0.18 | - | - |
| **CHEMOTHERAPY** |  |  |  |  |
| No | reference |  | reference |  |
| Yes | 0.08(0.05-0.14) | <0.0001 | 0.06(0.03-0.11) | <0.0001 |
| **SUBTYPE** |  |  |  |  |
| HoR+/HER2- | reference |  | reference |  |
| HoR+/HER2+ | 1.67(0.74-3.77) | 0.22 | 2.16(0.95-4.94) | 0.07 |
| HoR-/HER2+ | 2.97(1.46-6.03) | <0.01 | 2.75(1.30-5.81) | 0.01 |
| HoR-/HER2- | 3.41(1.82-6.39) | <0.0001 | 2.75(1.45-5.22) | <0.01 |
| **AGE (year)** |  |  |  |  |
| ＜60 | reference |  | reference |  |
| ≥60 | 1.95(1.13-3.37) | 0.02 | 1.33(0.75-2.36) | 0.32 |

Abbreviations: HR: hazard ratio; HoR: hormone receptor; HER‐2: human epidermal growth factor receptor‐2
